# Analysis of long non-coding RNAs highlights tissue-specific expression patterns and epigenetic profiles in normal and psoriatic skin

**DOI:** 10.1186/s13059-014-0570-4

**Published:** 2015-01-30

**Authors:** Lam C Tsoi, Matthew K Iyer, Philip E Stuart, William R Swindell, Johann E Gudjonsson, Trilokraj Tejasvi, Mrinal K Sarkar, Bingshan Li, Jun Ding, John J Voorhees, Hyun M Kang, Rajan P Nair, Arul M Chinnaiyan, Goncalo R Abecasis, James T Elder

**Affiliations:** Department of Biostatistics, Center for Statistical Genetics, School of Public Health, M4614 SPH I, University of Michigan, Box 2029, Ann Arbor, MI 48109-2029 USA; Michigan Center for Translational Pathology, University of Michigan Medical School, Ann Arbor, MI USA; Department of Dermatology, University of Michigan, Ann Arbor, MI USA; Ann Arbor Veterans Affairs Hospital, University of Michigan, Ann Arbor, MI USA; Department of Molecular Physiology and Biophysics, Center for Quantitative Sciences, Vanderbilt University, Nashville, TN USA; Laboratory of Genetics, National Institute on Aging, National Institutes of Health, Baltimore, MD USA; Department of Pathology, University of Michigan Medical School, Ann Arbor, MI USA; Department of Urology, University of Michigan Medical School, Ann Arbor, MI USA; University of Michigan Medical School, 7412 Medical Sciences Building 1, 1301 E. Catherine, Ann Arbor, MI 48109-5675 USA

## Abstract

**Background:**

Although analysis pipelines have been developed to use RNA-seq to identify long non-coding RNAs (lncRNAs), inference of their biological and pathological relevance remains a challenge. As a result, most transcriptome studies of autoimmune disease have only assessed protein-coding transcripts.

**Results:**

We used RNA-seq data from 99 lesional psoriatic, 27 uninvolved psoriatic, and 90 normal skin biopsies, and applied computational approaches to identify and characterize expressed lncRNAs. We detect 2,942 previously annotated and 1,080 novel lncRNAs which are expected to be skin specific. Notably, over 40% of the novel lncRNAs are differentially expressed and the proportions of differentially expressed transcripts among protein-coding mRNAs and previously-annotated lncRNAs are lower in psoriasis lesions versus uninvolved or normal skin. We find that many lncRNAs, in particular those that are differentially expressed, are co-expressed with genes involved in immune related functions, and that novel lncRNAs are enriched for localization in the epidermal differentiation complex. We also identify distinct tissue-specific expression patterns and epigenetic profiles for novel lncRNAs, some of which are shown to be regulated by cytokine treatment in cultured human keratinocytes.

**Conclusions:**

Together, our results implicate many lncRNAs in the immunopathogenesis of psoriasis, and our results provide a resource for lncRNA studies in other autoimmune diseases.

**Electronic supplementary material:**

The online version of this article (doi:10.1186/s13059-014-0570-4) contains supplementary material, which is available to authorized users.

## Background

Long non-coding RNAs (lncRNAs) have received much attention in the past several years. Coincident with improved annotation of functional elements [1,2], it has been appreciated that a large portion of the genome is transcribed during the course of development, much of which represents lncRNA [3]. LncRNAs resemble mRNAs because they are typically transcribed from active chromatin [4], polyadenylated, and capped; however, they do not direct protein synthesis [5]. LncRNAs play important functional roles in epigenetic regulation, by forming networks of ribonucleoprotein complexes with chromatin regulators [6], and targeting their action to appropriate genomic regions both in *cis* and in *trans* [5,7,8]. Initially recognized for their role in X-chromosome inactivation [9], lncRNAs are increasingly being implicated in a variety of disease states, including susceptibility to infection [10], neurodegenerative diseases [11], and cancer [12-15].

Recently, discovery and analysis of non-coding RNAs have been enhanced by RNA-seq technology, and different pipelines have been developed to identify novel lncRNAs using RNA-seq data [1,13,16,17]. However, despite successful studies highlighting important roles of lncRNAs in different tissues and diseases [12,13,15,18], little is known about the roles of lncRNAs in human autoimmune diseases [19]. Furthermore, biological inference of lncRNA function remains a challenging task, given their currently-limited annotation status and low expression levels [3].

Psoriasis is a chronic immune-mediated inflammatory and hyperproliferative disease of skin and joints, affecting around 2% of the population [20,21]. Recently, we [22] and others [23] have applied RNA-seq technology to the analysis of protein-coding genes in psoriatic lesions, compared to uninvolved skin from the same individual [23], or to normal skin [22]. Aided by the availability of large numbers of samples, we identified multiple networks of coordinately expressed genes in normal versus psoriatic skin utilizing weighted gene co-expression network analysis [22]. Moreover, we found strong enrichment for genes that are known targets of lncRNA-mediated terminal differentiation in both normal and psoriatic skin [22]. In the skin, several lncRNAs have been shown to play key roles in both epidermal specification during development [6] and epidermal terminal differentiation [18]. A lncRNA named *PRINS* (Psoriasis susceptibility-related RNA Gene Induced by Stress) has also found to be essential in the survival of keratinocytes under stress condition and may contribute to psoriasis susceptibility [24]. These observations motivated us to consider psoriasis as an exemplar for the roles of lncRNAs in autoimmune disease.

To accomplish this, we first applied a computational approach [13] and stepwise filtering procedures to identify high-confidence lncRNAs expressed in the RNA-seq cohort. We then developed an analytical pipeline to globally characterize skin-expressed lncRNAs, with a particular focus on novel lncRNAs that were not previously annotated. We tailored our analysis to ask: (i) whether newly identified lncRNAs would have different expression behaviors compared to mRNAs and previously-annotated lncRNAs; and (ii) whether we could use existing biological information and data to infer the functional roles of the identified lncRNAs. Applying the aforementioned tools, we identified tissue-specific expression patterns and epigenetic profiles for novel lncRNAs, which are more pronounced than for previously-annotated (known) lncRNAs, with a significantly higher proportion of novel lncRNAs differentially expressed in lesional psoriatic skin. By examining patterns of co-expression with mRNAs of known function, we found strong enrichment for immune-related functions among all identified lncRNAs, particularly those that are differentially expressed in psoriatic skin. Overall, our study highlights the importance of lncRNAs in the pathogenesis of psoriasis, and provides a valuable resource for lncRNA studies in other autoimmune diseases.

## Results

### Identification of lncRNAs in normal, uninvolved, and lesional psoriatic skin

**Figure 1:**
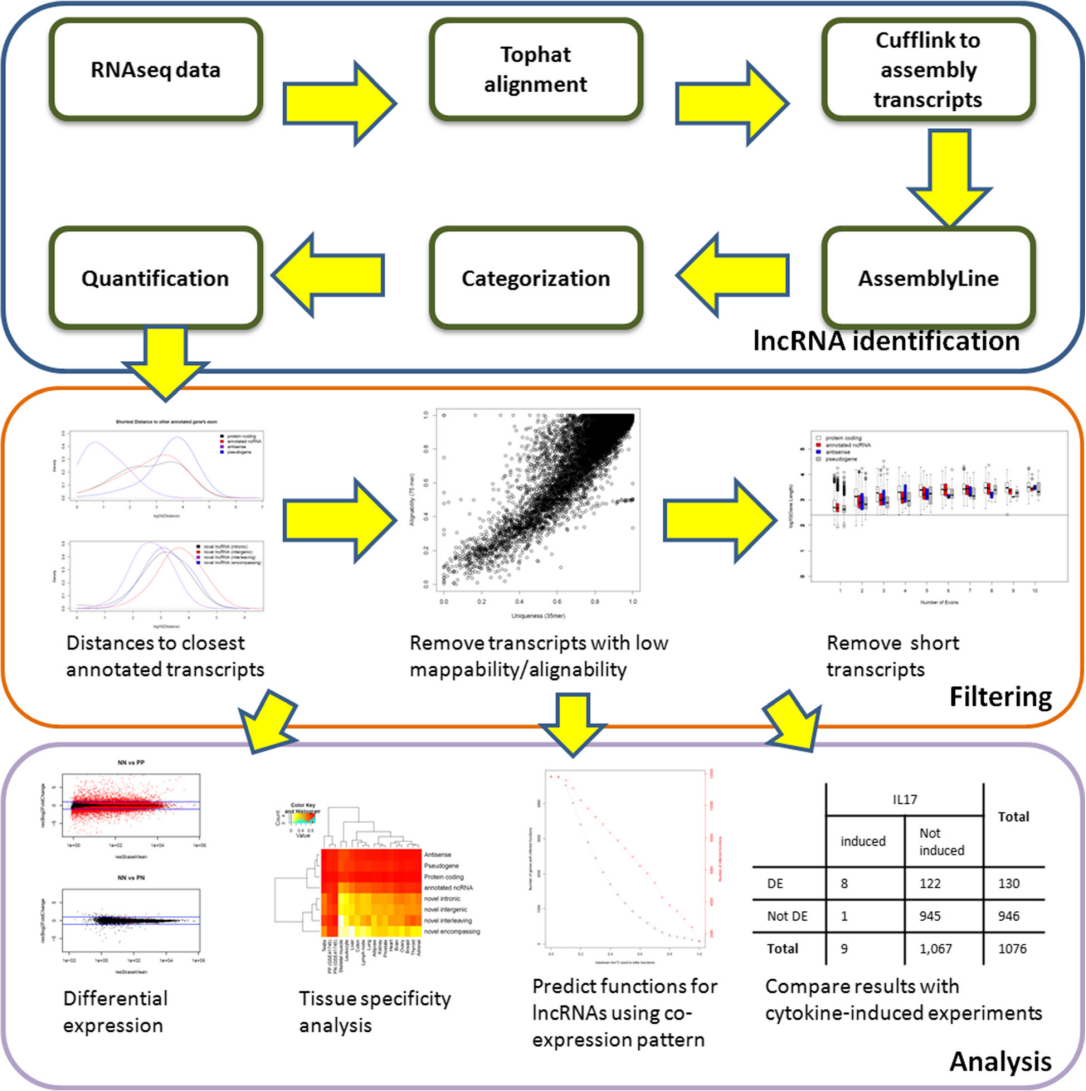
**Overview of the analysis pipeline.** We first performed Tophat alignment and identified uniquely mapped reads for each RNA-seq sample, we then assembled the transcripts using Cufflinks for each sample. We used a computational approach to nominate potential novel transcripts (Prensner JR *et al.*, [13]) by comparing with Ensembl gene set. We removed those potential novel transcripts which are close (that is, <2 kb) to any exons from any annotated transcripts, inhabited in regions with lower mappability/alignability, or less than 200 bp in length. We quantified the gene expressions using read counts. We then normalized the values across the samples and performed differential expression analysis using DESeq. We inferred the properties and biological functions of the lncRNAs by comparing results with other RNA-seq experiments and using co-expression analysis.

We analyzed high-throughput sequencing data from polyA+ RNA-derived cDNA (designated as RNA-Seq) from 216 skin samples (99 lesional psoriatic (PP), 27 uninvolved psoriatic (PN), and 90 normal controls (NN); see Additional file 1 for additional information regarding the patient cohort). The dataset consisted of 174 samples that have been previously described (92 PP and 82 NN) [22] and 42 new samples (7 PP, 27 PN, and 8 NN), with each of the PN samples being paired with one PP sample (on average 40 million reads per sample). An overview of the analysis pipeline is shown in Figure 1. We used Tophat [25] to align reads to the human genome, utilizing only uniquely mapped reads, followed by Cufflinks [26] to identify transcripts in each sample, using *ab initio* assembly. To detect unannotated transcripts, we employed a computational approach [13] that combines information (such as evidence for recurrent expression and percentile of abundance) across all the samples in the dataset. Using Ensembl version 74 as reference [27], identified transcripts were classified into five different categories (Additional file 2): (i) protein-coding; (ii) pseudogene; (iii) annotated ncRNA; (iv) antisense; (v) unannotated (novel) transcripts. The novel transcripts were further divided into: (a) novel intronic (unannotated transcript with exon(s) in intronic region(s) of the reference gene); (b) novel intergenic (unannotated transcript with exon(s) in intergenic regions defined by the reference); (c) novel interleaving (exons of the unannotated transcript in intronic and intergenic space relative to reference); and (d) novel encompassing (exons from reference gene(s) in the intronic space of the unannotated transcript).

As the identification of unannotated transcripts could be due to artifacts involving mappability or artifactual nomination of immature mRNA fragments, we applied several filtering steps to remove potential artifacts (Materials and methods; Figure 1). First, we identified transcripts with coverage in at least 5% (that is, ≥11) of the samples in our full dataset. Next, we removed false lncRNA predictions due to premature mRNA fragments by calculating, for each annotated transcript, the genomic distance from its closest annotated gene exon (Additional file 3: Figure S1) and removing unannotated transcripts which map within 2 kb of the nearby annotated exons. Next, we imposed mappability and length filters to remove unannotated transcripts in genomic regions of low complexity and with short lengths (<200 bp) [3]. These filters are described in more detail in the Materials and methods and in Additional file 3: Figures S2-5. In subsequent analyses, we only considered transcripts with ≥1 read per sample on average. An expressed transcript identified in this study is thus defined as a transcript that passes the above quality control filters.

**Figure 2:**
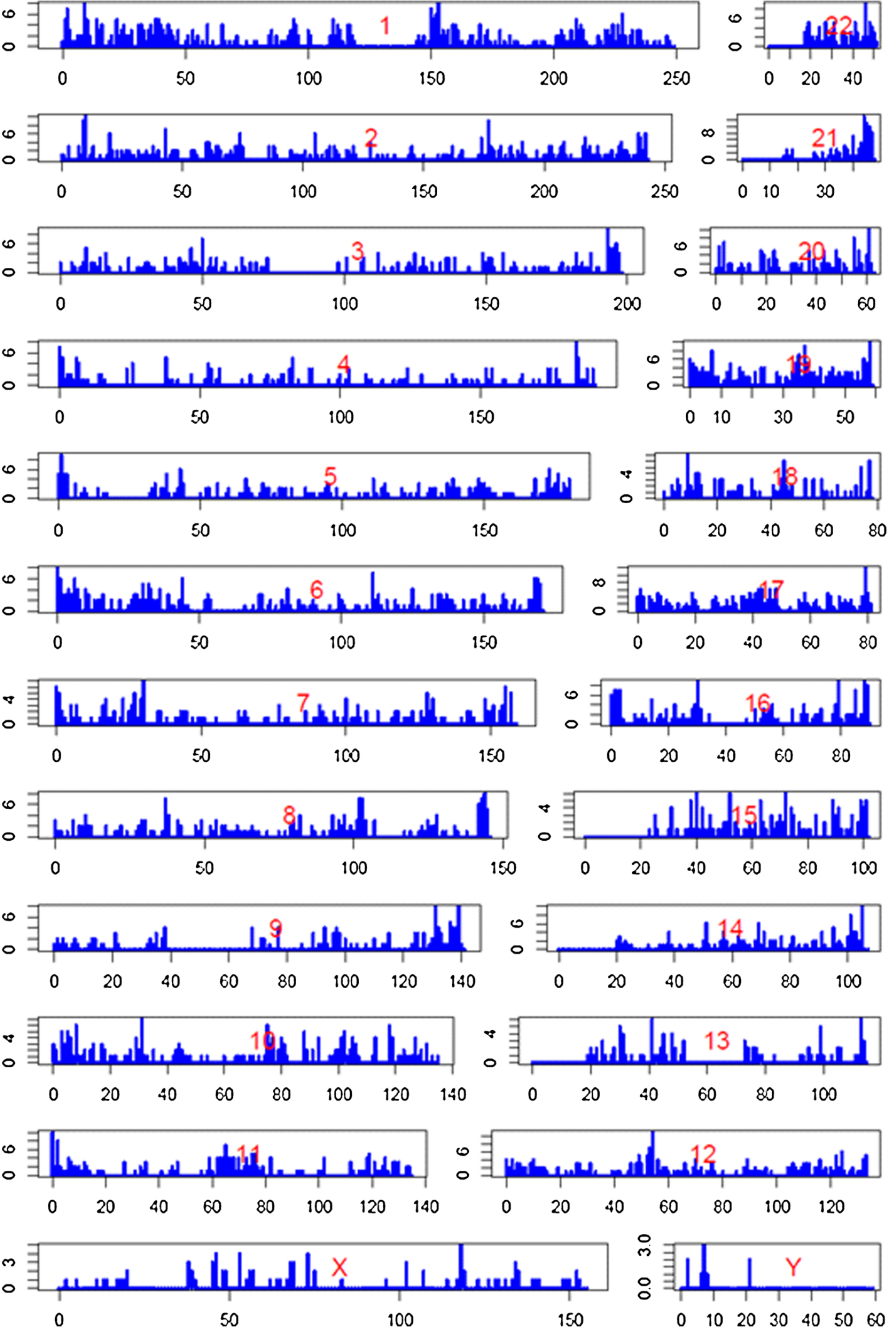
**Genomic map of lncRNAs expressed in skin tissues across the genome.** The number of lncRNAs identified in this study (y-axis) per megabase across the genome (x-axis).

Table 1 shows the number of transcripts remaining after the aforementioned filtering steps (information regarding individual transcripts in Additional file 4). We identified 4,022 expressed lncRNAs, of which 2,942 (73%) were previously annotated and 1,080 (27%) were novel. The genomic distribution of expressed lncRNAs is shown in Figure 2, and a corresponding density map for novel lncRNAs is shown in Additional file 3: Figure S6. On average, we detected 1.3 expressed lncRNAs (annotated + novel) per megabase (Mb) across the genome (approximately 0.4 novel lncRNAs/Mb). We evaluated the protein coding potential of the identified novel lncRNAs and only two of them were predicted to be candidate coding transcripts, comparable to results reported previously [13] (Additional file 3: Figure S7). Moreover, in agreement with previous studies [3,28], the identified lncRNAs in our dataset tend to have fewer exons than protein-coding genes (that is, around 70% with fewer than 3 exons, Additional file 3: Figure S8).

**Table 1:** **Transcripts remaining after application of various filtering steps**

					**Novel**			
**Filter**	**Protein coding**	**Antisense**	**Pseudogene**	**Annotated lncRNA**	**Intronic**	**Intergenic**	**Interleaving**	**Encompassing**
Raw	16,246	2,902	3,670	4,593	823	1,825	142	113
≥5% (11) samples	16,225	2,897	3,650	4,585	822	1,820	141	112
Distance (≥2 kb)	16,225	2,897	3,650	4,585	336	1,269	22	46
Mappability	14,468	2,338	1,698	3,646	249	993	18	36
Length (≥200 bp)	14,461	2,336	1,693	3,642	244	984	18	36
≥216 mapped reads	14,011	2,294	1,476	2,942	196	840	15	29

To evaluate the efficiency of the lncRNA discovery pipeline we used a subset of the data (that is, the 174 samples described in [22]) to identify novel lncRNAs using the procedures described above, and asked whether the identified transcripts are expressed in the remaining 42 independent samples. We found that over 95% of the novel lncRNAs identified in these 174 samples were also expressed in the 42 independent samples (Additional file 3: Figure S9). The result illustrates the robustness of the pipeline and the filtering procedures in the identification of previously unannotated lncRNAs.

### Differences in gene expression patterns between novel lncRNAs and annotated transcripts

**Figure 3:**
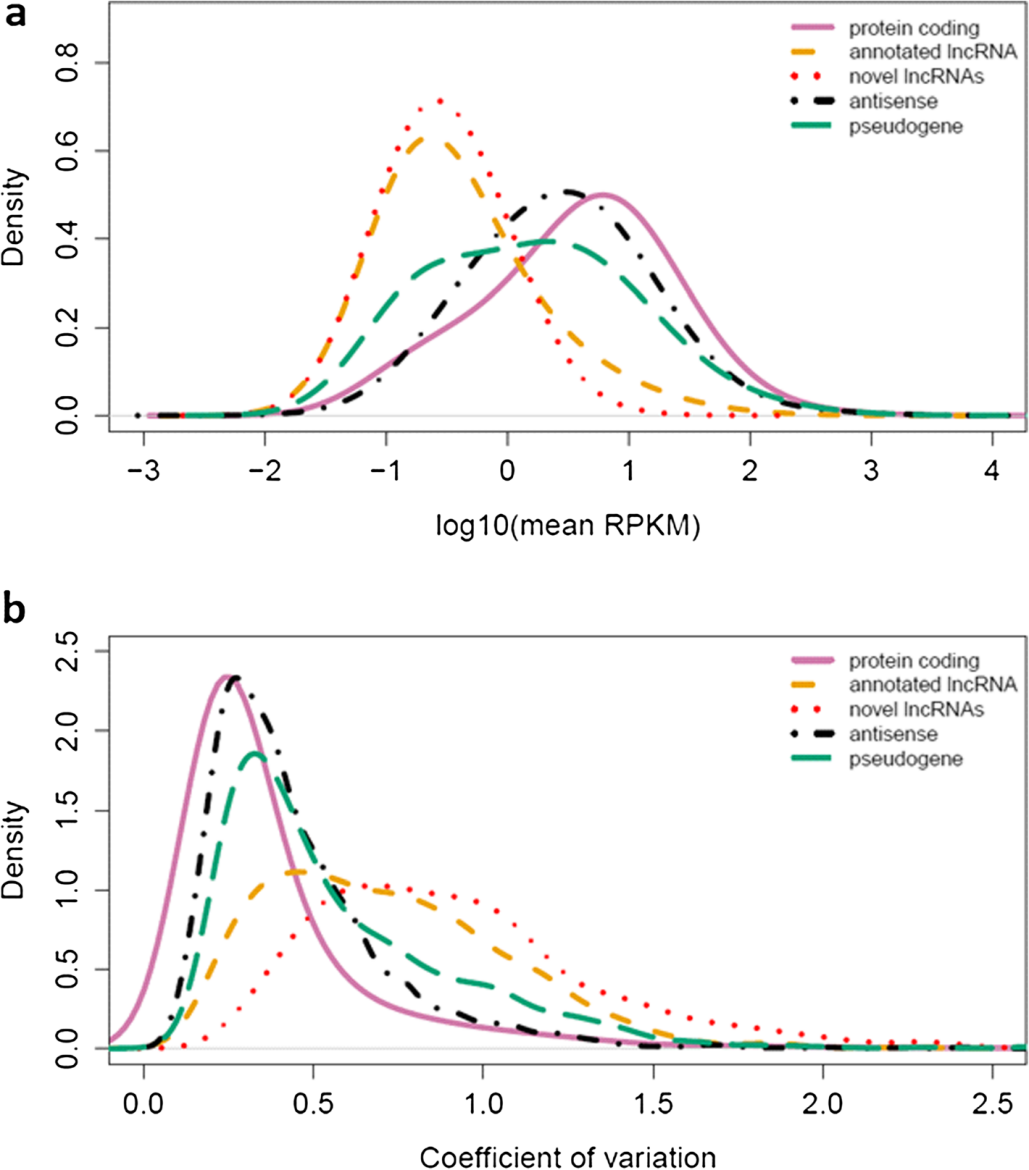
**Expression behaviors for different gene categories.** The mean gene expression in RPKM is shown in **(a)** and coefficient of variation in the normal skin samples for different transcript categories is shown in **(b)**.

In agreement with previous studies [3,29], our results indicate that lncRNAs tend to be expressed at lower levels and have higher coefficients of variation than other gene categories (that is, protein-coding, pseudogene, antisense) (Figure 3). Notably, expression of novel lncRNAs as a group was significantly lower than observed for annotated protein-coding genes (*P* <2.2 × 10^−16^) and previously described lncRNAs (*P* = 9.4 × 10^−4^ by Mann–Whitney U test). Using only normal skin samples, the novel lncRNAs also exhibited significantly larger coefficients of variation than both the annotated protein-coding genes (*P* <2.2 × 10^−16^) and lncRNAs (*P* <2.2 × 10^−16^).

Because our results identified differences in expression patterns between novel lncRNAs and other gene categories, we next examined their respective expression profiles in PP, PN, and NN skin. Using the normalization procedure and negative binomial test implemented in DESeq [30], we performed three differential expression analyses (that is, NN vs. PP; NN vs. PN; and PN vs. PP). Using the criteria of false discovery rate (FDR) ≤0.1 and |log_2_ fold change (FC)| ≥1 to declare significance, we identified 4,102 differentially expressed genes (DEGs) when comparing NN versus PP skin, including 1,214 lncRNAs (709 annotated and 505 novel) (Table 2 and Additional file 5). We observed similar proportions of DEGs for protein-coding genes and known lncRNAs (17% to 24%). As expected, the results for protein-coding genes are consistent with our earlier study using a subset of these samples [22]. Compared to known lncRNAs, the novel lncRNAs had a strikingly and significantly (Fisher’s Exact test: *P* = 7 × 10^−103^) higher proportion (47%) of differentially expressed transcripts in PP vs. NN skin (for example, 41% for intronic and 47% for intergenic). Moreover, two of the most significantly differentially-expressed lncRNAs were novel, one being downregulated (annotated here as ‘G25746’: *P* <1 × 10^−323^; FC = 0.02; see Additional file 4 for detailed annotations for the novel lncRNAs), and the other being an upregulated (annotated here as ‘G2608’: *P* = 4.7 × 10^−129^; FC = 81.20) (Additional file 3: Figure S10) transcript that mapped to the epidermal differentiation complex (EDC) on chromosome 1q21.3 [31]. As expected, using the chosen significance criteria, we observed very few differences between NN and PN skin (three differentially expressed transcripts), supporting results from a previous study on protein-coding transcripts, which, using slightly more lenient criteria, found that fold changes in expression level between NN and PN transcripts were in the range of 1.3 to 1.9 [32]. As expected given the similarity of NN and PN transcriptomes, we observed similar proportions of differentially expressed transcripts (that is, approximately 40% for novel lncRNAs) in the PP vs. PN comparisons as in the PP vs. NN comparisons (Table 2). Since novel lncRNAs tend to have lower expression levels (Figure 1), we examined if transcripts with low expression tended to be more differentially expressed according to the negative binomial test, and we did not find any significant associations within the novel lncRNA nor the annotated transcript categories (Additional file 3: Figure S11). We then performed qRT-PCR experiments on 18 independent skin samples (6 PP; 6 PN; and 6 NN) to assess the differential expression for lncRNAs G2608, G25746, and G36220 (a differentially expressed lncRNA (ENSG00000237499) in psoriatic skin and located within a psoriasis susceptibility locus *TNFAIP3*). All showed significant results (*P* <0.05) and were in excellent agreement with the RNA-seq findings (Additional file 3: Figure S12).

**Table 2:** **Numbers and proportions (in percentage) of differentially expressed genes for different gene categories under three different comparisons: normal vs. lesional psoriatic skin (NN vs. PP), uninvolved vs. lesional psoriatic skin (PN vs. PP), and normal vs. uninvolved skin (NN vs. PN)**

					**Novel**				
**No. DEGs (%)**	**Protein coding (n = 14,011)**	**Antisense (n = 2,294)**	**Pseudogene (n = 1,476)**	**Annotated lncRNA (n = 2,942)**	**Total (n = 1,080)**	**Intronic (n = 196)**	**Intergenic (n = 840)**	**Interleaving (n = 15)**	**Encompassing (n = 29)**
NN vs. PP	2,342	408	138	709	505	81	396	9	19
	(17%)	(18%)	(9%)	(24%)	(47%)	(41%)	(47%)	(60%)	(66%)
PN vs. PP	2,146	369	161	613	436	76	337	8	15
	(15%)	(16%)	(11%)	(21%)	(40%)	(39%)	(40%)	(53%)	(52%)
NN vs. PN	3	0	0	0	0	0	0	0	0
	(0.03%)	(0%)	(0%)	(0%)	(0%)	(0%)	(0%)	(0%)	(0%)

**Figure 4:**
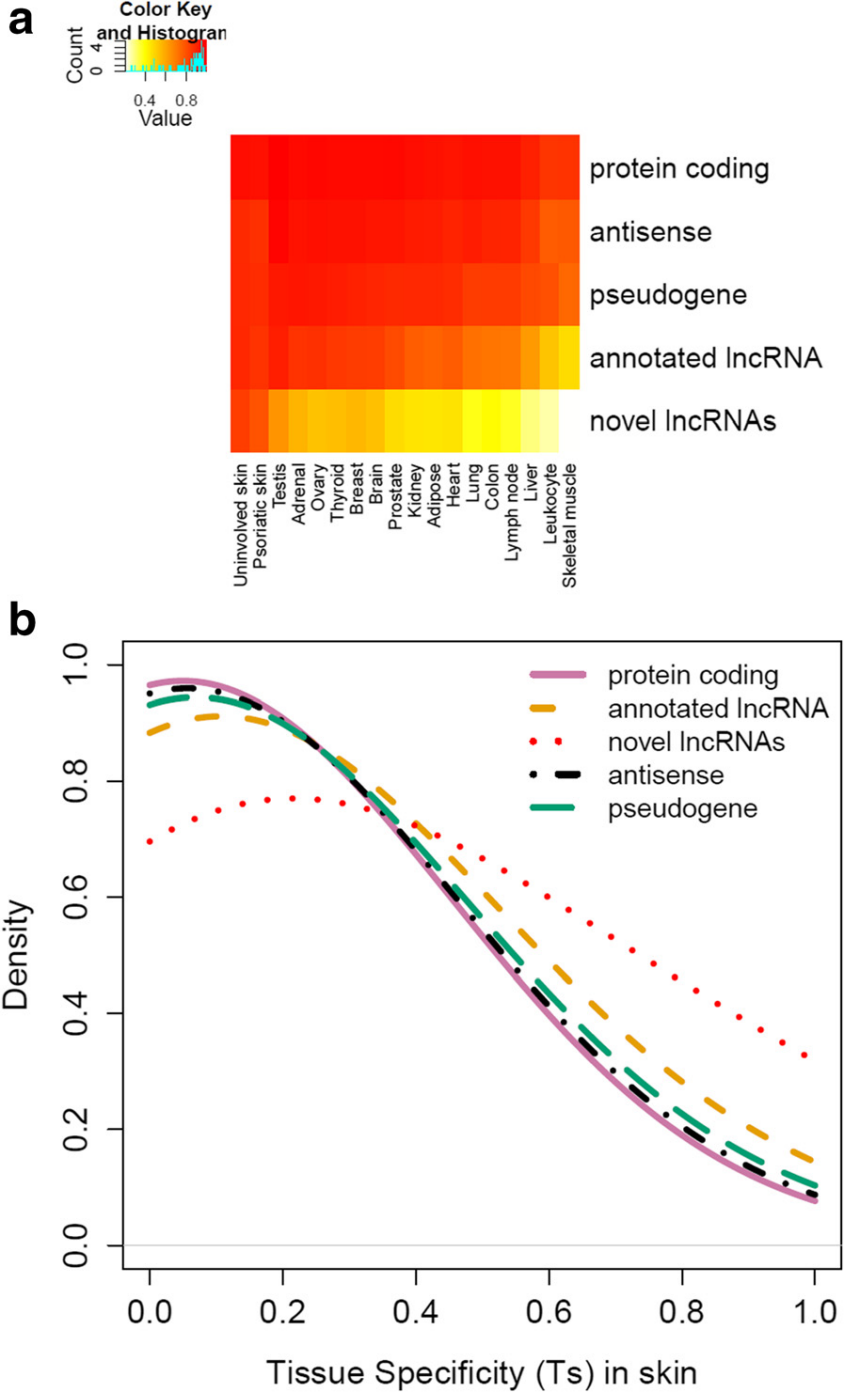
**Tissue specificity analysis for different gene categories. (a)** Heatmap showing the proportion of genes from each category expressed in different tissue types. **(b)** Tissue specificity (*T*_*s*_) for different gene categories in skin when comparing with 16 other tissue types.

To further explore the enrichment of novel lncRNAs, we evaluated the proportions of expressed transcripts and the tissue-specificity index (*T*) for our identified genes in 17 different tissues using two publicly available RNA-seq datasets: the Body Map 2.0 project (which includes data for 16 different tissues) [33] and a study of three PN/PP biopsy pairs [23]. While the proportions of expressed protein-coding genes are similar across different tissues, the proportions of expressed lncRNAs we identify are higher in skin (PP/PN) than other tissues [3] (Figure 4). This contrast is notably more pronounced for the novel lncRNAs, in that a much lower proportions are annotated as expressed in tissues other than skin (Figure 4a). We next computed the skin specificity index (*T*_*s*_, see Materials and methods) using gene expression levels across the 17 different tissues. The results (Figure 4b) further support our hypothesis that the novel lncRNAs tend to have higher expression levels in skin than in other tissues. In fact, the skin specificity index for novel lncRNAs is significantly (*P* <2.2 × 10^−16^ by Mann–Whitney U test) higher than those for annotated lncRNA and protein-coding transcripts.

**Figure 5:**
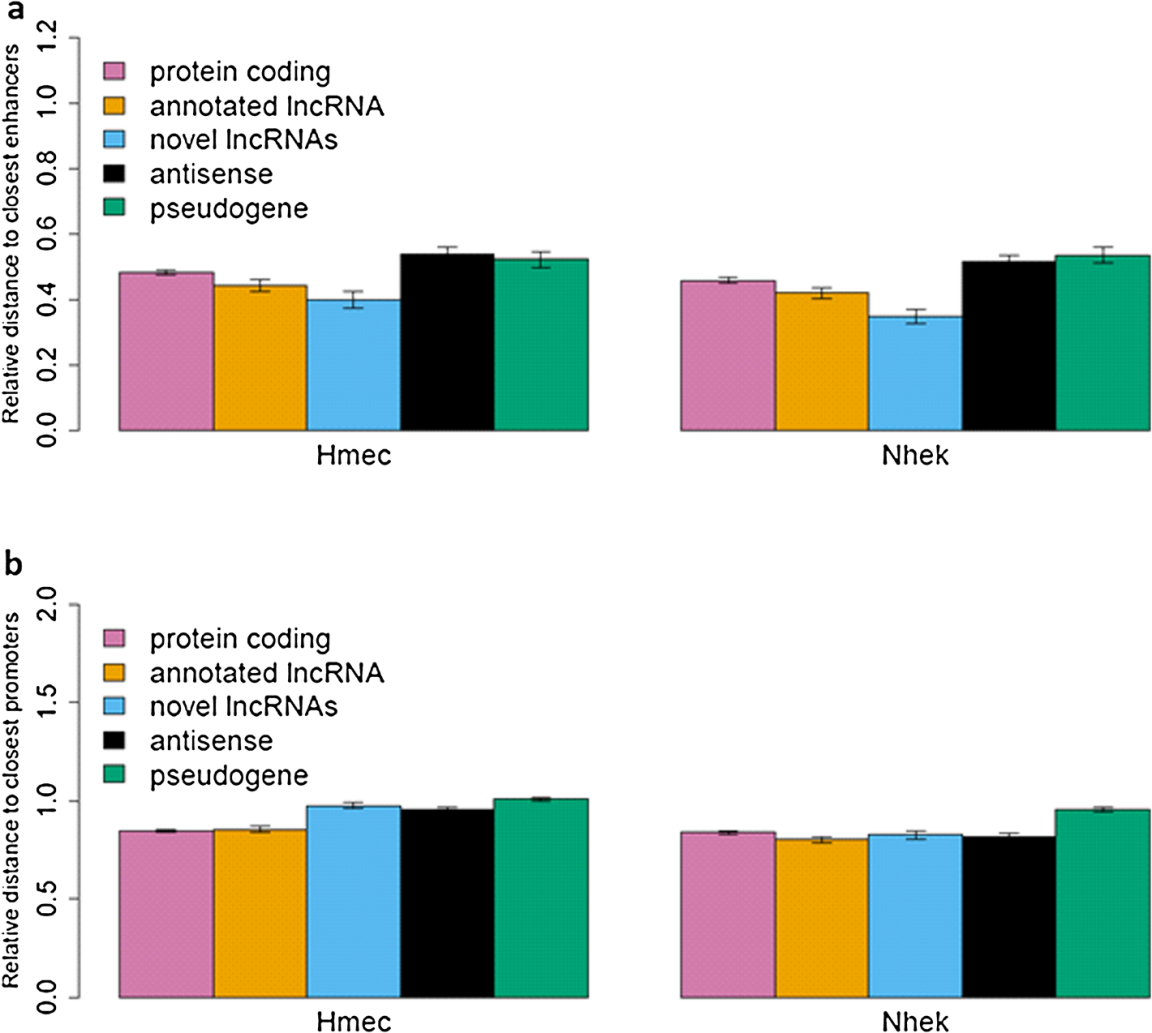
**Relative distance to enhancers (a) and promoters (b) for different transcript classes.** The means and error bars depict the relative distance (*D*_*ecto*_ */D*_*average*_) to the enhancer **(a)** and promoter **(b)** elements for genes in each category in these two ectodermally derived cell types (HMEC and NHEK). *D*_*ecto*_ is the closest distance to the enhancer (or promoter) in NHEK (or HMEC), and *D*_*average*_ is the average closest distance to the enhancer (or promoter) to the other cell types.

To address whether the tissue-specific expression pattern of the novel lncRNAs correlates with distinct epigenetic marker profiles in skin, we identified and measured the distances between the genomic location of each transcript and the nearest predicted enhancer or promoter element in nine different ENCODE cell lines [34,35]. We then computed the relative distance (*D*_*ecto*_*/D*_*average*_) to the nearest element, comparing the distance to closest enhancer (or promoter) in the ectodermally-derived cell type (*D*_*ecto*_), to the distance to the closest enhancer (or promoter) element in the other eight cell types (*D*_*average*_, see Materials and methods). We used relative distance instead of absolute distance to avoid bias due to systematic differences in the proximity to transcriptional regulatory elements between different transcript categories. Moreover, because different cell lines tend to have different numbers of predicted enhancer/promoter elements, the ratio of D_ecto_/_Daverage_ would not necessarily be 1. Specifically, there are higher numbers of predicted enhancers in ectodermally derived cell types (86,259 for NHEK and 72,108 for HMEC) when compared to other cell types (59,492 on average). Figure 5 illustrates that genes tend to be closer to enhancer elements in the ectodermally-derived NHEK (normal human epidermal keratinocytes) and HMEC (human mammary epithelial cells) lines than in other cell types in general (that is, relative distance <1). However, compared to annotated transcripts (that is, protein-coding genes and annotated lncRNAs), the relative distances to enhancers in ectodermally-derived cell types are significantly shorter for novel lncRNAs (*P* = 8 × 10^−4^ in HMEC and *P* = 1.6 × 10^−9^ in NHEK).

Previous studies have noted that lncRNAs can be classified as enhancer-associated (elncRNAs) or promoter-associated (plncRNAs) [36-38]. In an effort to understand the potential impact or effect of these elncRNAs/plncRNAs on neighboring genes, we first determined the candidate elncRNAs or plncRNAs using predicted enhancers/promoters in NHEK as a reference. The elncRNAs were identified as having close proximity (<5 kb) with enhancers but not with promoters. Conversely, the plncRNAs were identified as being in close proximity (<5 kb) to promoters but not to enhancers. This analysis identified 764 elncRNAs (483 annotated and 281 novel) and 369 plncRNAs (342 annotated and 27 novel), resulting in a substantially higher proportion of novel lncRNAs among the elncRNAs than among the plncRNAs (full list in Additional file 6). Additionally, the skin specificity index/FC value (in the PP vs. NN comparison) obtained from the elncRNAs is significantly correlated (by Spearman’s correlation test) with that obtained from the protein-coding genes neighboring the elncRNAs (G^elncRNAs^); similar results were obtained for the plncRNAs and the protein-coding genes neighboring the plncRNAs (G^plncRNAs^). Specifically, significance of the correlation in skin specificity index between elncRNAs and G^elncRNAs^ was *P =* 1.5 × 10^−3^ (ρ = 0.4), and between plncRNAs and G^plncRNAs^ was *P =* 5.3 × 10^−5^; ρ = 0.3); and the significance of correlation in FC in the PP/NN comparisons was *P =* 1.5 × 10^−6^ (ρ = 0.6) for elncRNAs vs. G^elncRNAs^ and *P =* 3 × 10^−8^ (ρ = 0.4) for plncRNAs vs. G^plncRNAs^. In contrast, we did not identify any difference when comparing the G^elncRNAs^ to G^plncRNAs^ (*P* >0.05). Furthermore, we observed a significant (*P* = 2.9 × 10^−4^) negative correlation between the skin specificity index (*T*_*s*_) of the annotated gene with its distance to the closest novel lncRNA (distance restricted to ≤1 Mb). Taken together, our results show that while both elncRNA and plncRNAs exhibit tissue specificity and differential expression profiles that are similar to their corresponding neighboring protein-coding genes, novel lncRNAs are more likely to be enhancer-associated than promoter-associated. These results further highlight the potential biological and functional roles of the novel lncRNAs, which constitute more than 37% of the elncRNAs we identified.

### Functional characterization of the identified lncRNAs

To better understand the functional ramifications of the expressed lncRNAs in our dataset, we deployed an analytical pipeline assessing the relationship of the identified lncRNAs to: (i) known psoriasis susceptibility loci; (ii) co-expressed annotated mRNAs; and (iii) lncRNAs expressed by cytokine-stimulated keratinocytes. We first asked whether the expressed lncRNAs are enriched in regions of biological interest in the cutaneous context. Notably, two of the regions of highest lncRNA densities (Figure 2) were the Epidermal Differentiation Complex (EDC, chromosome 1q21.3, 150–155 Mb) [39,40] and the Major Histocompatibility Complex (MHC, chromosome 6p21.3, 26–34 Mb) [41], both of which contain psoriasis-associated genes [42]. In the EDC, we identified 16 annotated and 12 novel lncRNAs, yielding a significant enrichment for novel lncRNAs mapping to the EDC when using annotated lncRNAs as background (*P* = 3 × 10^−2^, hypergeometric test). In contrast, for the MHC region, the enrichment of novel lncRNAs is not significant (20 annotated vs. 6 novel lncRNAs, *P* = 0.92) (see Discussion).

We next estimated the density of lncRNAs within known psoriasis susceptibility regions [42], and provided a comprehensive catalog of expressed lncRNAs in the psoriasis loci in Additional file 7 (Table S6). We identified 103 expressed lncRNAs (31 of them novel), and 26 of them were differentially expressed in PP vs. NN skin. Moreover, we found that the psoriasis locus 9q31.2, which has no expressed protein-coding gene, contains two expressed lncRNAs (see Discussion).

Typically lncRNAs are not functionally well-characterized, as only 59 of the 2,942 annotated lncRNAs identified in our sample had at least one functional annotation in the Gene Ontology or KEGG gene annotation databases [43,44], as opposed to 12,770 out of the 14,011 identified mRNAs. Because genes with similar co-expression patterns tend to exhibit functional coherency ([45] and Additional file 3: Figure S13), we utilized the co-expression patterns of mRNAs and lncRNAs to infer biological functions for the latter. For each lncRNA, we recorded the functional annotations corresponding to the most correlated gene(s), and we used a supervised approach to estimate the minimum correlation criteria (see Materials and methods; Additional file 3: Figures S13-15). Using squared Spearman’s rank correlation coefficient ≥0.5 as our criterion, we inferred biological functions for 959 lncRNAs (24% of all identified lncRNAs), including 490 differentially expressed lncRNAs (40% of all differentially expressed lncRNAs). Over 28% of the co-expressed pairs we identified are cis-pairs (within 1 Mb), suggesting many of these pairs may share similar regulatory mechanisms (Additional file 8: Table S7). We then identified the inferred functions that were enriched among the lncRNAs differentially expressed between normal and psoriatic skin (most significant results shown in Table 3; full results shown in Additional file 9: Table S8). These included functions related to cell-cell signaling, inflammation, and lipid metabolism, including ‘extracellular region’, ‘cytokine activity’, and ‘lipid metabolic process’. We also observed similar results for the novel lncRNAs (Table 3).

**Table 3:** **Enriched (FDR ≤ 0.1) inferred functions among all the differentially expressed lncRNAs (DE lncRNAs) and differentially expressed novel lncRNAs (DE novel lncRNAs) in psoriatic skin**

**Enrichment**		**No. of genes with the function**	**No. of DEGs with the function**	***P*** **value**	**FC**	**FDR**
**DE lncRNAs (total)**	**Cytokine activity**	42	41	5.28E-12	1.91	3.72E-08
	**Extracellular space**	69	60	4.96E-11	1.70	3.50E-07
	**Extracellular region part**	87	72	7.77E-11	1.62	5.48E-07
	**Cellular lipid metabolic process**	68	56	1.78E-08	1.61	1.25E-04
	**Fatty acid metabolic process**	42	37	1.50E-07	1.72	1.05E-03
	**Lipid metabolic process**	87	66	4.21E-07	1.48	2.96E-03
	**Monocarboxylic acid metabolic process**	46	39	6.41E-07	1.66	4.51E-03
	**Lipid biosynthetic process**	45	38	1.11E-06	1.65	7.80E-03
	**Regulation of inflammatory response**	23	22	1.90E-06	1.87	1.34E-02
	**Cytokine receptor interaction**	34	30	2.18E-06	1.73	1.54E-02
**DE novel lncRNAs**	**Extracellular space**	69	30	3.99E-07	2.33	1.32E-03
	**Extracellular region part**	87	34	1.42E-06	2.09	4.72E-03
	**Cytokine activity**	42	20	6.80E-06	2.55	2.26E-02
	**Cellular lipid metabolic process**	68	27	1.34E-05	2.13	4.46E-02
	**Lipid metabolic process**	87	32	1.42E-05	1.97	4.72E-02
	**Biosynthesis of unsaturated fatty acid**	6	6	1.97E-05	5.36	6.54E-02
	**Exidoreductase activity**	48	21	2.14E-05	2.34	7.09E-02

FC refers to the observed to expected ratios for the enrichment. For illustration purposes, only inferred functions annotated with at most 100 genes are shown.

Interleukin-17 (IL-17) and tumor necrosis factor (TNF) are key proinflammatory cytokines in psoriasis [21], and we have recently shown that the RNA-seq based psoriatic skin transcriptome manifests a significant IL-17 stimulation signature [22]. To explore the effects of these key cytokines on lncRNA expression profiles, we utilized an RNA-seq dataset generated to characterize the transcriptome of keratinocytes stimulated for 24 h with IL-17 and TNF. We evaluated whether the cytokine-enhanced or cytokine-repressed lncRNAs would be enriched among lncRNAs that are up- or downregulated in the psoriatic skin (cytokine-enhanced and cytokine-repressed lncRNAs are shown in Additional file 10: Table S9). Consistent with the above findings showing enrichment of immune-related functions for the identified lncRNAs, the upregulated lncRNAs in psoriatic skin were significantly enriched for cytokine-stimulated lncRNAs induced by IL17 + TNF treatment of keratinocytes (*P* <3.3 × 10^−2^ after multiple testing correction, and observed-to-expected ratio >2; Additional file 11: Table S10). We did not identify any enrichment for downregulated lncRNAs among cytokine-repressed transcripts, whether annotated or novel.

## Discussion

Previous transcriptomic studies have emphasized the analysis of protein-coding transcripts, using mRNA expression levels to characterize the patterns and potential functional roles of their translated proteins [3]. The development of next generation sequencing technology has greatly accelerated the discovery and characterization of a new class of biologically-significant RNA transcripts - the lncRNAs [46,47]. As a class, lncRNAs tend to be under less stringent evolutionary constraint, to be expressed at lower levels, and are preferentially enriched in the nucleus [3]. Moreover, the structural rules governing lncRNA function are just now beginning to come to light [48].

Using a computational approach [13], we first enumerated known and novel lncRNAs in biopsies of normal (NN) skin from healthy controls, from lesional psoriatic (PP) skin, and in a subset of 27 affected individuals, from paired biopsies of uninvolved psoriatic (PN) skin. The ENCODE project identified 9,000 lncRNA gene in the human genome [1,3], and we could detect expression of approximately 40% of these in our skin samples. We further identified over 1,000 novel lncRNAs in our data, many of which were not well-expressed in other tissue types (Figure 4). Based on this finding, we would speculate that many more tissue-specific lncRNAs remain to be identified in other tissue/cell types.

A notable feature of the novel lncRNAs we identified is their tissue specificity. We assessed this parameter by comparison to lncRNAs we identified in data generated by the BodyMap 2.0 project [33] as well as in an independent skin RNA-seq sample [23] (Figure 4). In addition to protein-coding transcripts, antisense and pseudogene transcripts manifested substantial overlap between expression in skin and other tissues. While this overlap was somewhat less for known lncRNAs, these four transcript classes were clearly distinguished from the novel lncRNAs. Taken together with the observations that lncRNAs play crucial roles in both the development (ANCR) [6] and the terminal differentiation of skin (TINCR) [18], these results further suggest important, tissue-specific roles for the identified lncRNAs in skin development and differentiation. Indeed, seven lncRNAs (three of them novel) were shown to be highly correlated in their expression (that is, ρ ≥0.7) with genes in the differentiation-associated clusters identified in our previous study of protein-coding genes (annotated as N15 and P23 in [22]). To our knowledge, this is the first study to show that novel lncRNAs identified in a differentiated tissue behave substantially differently than do annotated lncRNAs in terms of tissue specificity in both the transcriptomic and epigenetic scales. More complete analysis of other tissues will be needed to determine whether this conclusion can be extended to tissue-specific versus more widely expressed lncRNAs.

In an effort to better understand the biological significance of the novel lncRNAs, we took a clue from a recent study comparing intergenic lncRNAs arising from enhancer-associated elements (elncRNAs) to those arising from promoter-associated elements (plncRNAs) in mouse erythroblasts [38]. Utilizing a panel of nine cell types in which promoters and enhancers have been well-mapped in ENCODE [34,35], we found that novel lncRNAs mapped significantly closer to enhancer sites in the two ectodermally-derived cell lines (namely human mammary epithelial cells (HMEC) and normal human epidermal keratinocytes (NHEK), relative to the other seven non-ectodermally-derived lines. In contrast, there was no significant difference in the relative distance to promoter elements in these comparisons. Both enhancer-associated and promoter-associated lncRNAs were highly correlated with the expression of nearby protein-coding genes. Based on these findings, we would speculate that the novel lncRNAs identified in this study may participate in the control of tissue-specific gene expression.

We explored the potential functions of the identified lncRNAs by correlating their co-expression patterns across combined NN and PP samples with those of known, biologically-annotated mRNA transcripts. This analysis identified enriched functions consistent with those identified in previous transcriptome analyses of psoriasis that were focused on protein-coding genes [22,49-55]. Furthermore, the novel lncRNAs that are differentially expressed yielded higher observed to expected ratios for the enrichment of inferred immunological functions, compared to all of the differentially expressed lncRNAs as a whole (Table 3). When taken together with the higher percentage of differentially-expressed novel lncRNAs compared to differentially expressed known lncRNAs (Table 2), these observations suggest that the novel lncRNAs we have identified may exert important biological functions in psoriatic skin.

Compared to known lncRNAs, significantly more of the novel lncRNAs were differentially expressed in psoriasis (Table 2). While intriguing, this result needs to be interpreted with caution for several reasons, including potential differences in RNA preparation and purity across studies, tissue-dependent expression, as well as differential overall expression of known vs. novel lncRNAs. However, we evaluated the latter possibility and found no significant difference in the distribution of absolute expression levels compared to extent of differential expression in PP versus NN skin for either annotated or novel lncRNAs. When we imposed a minimum median RPKM (among cases or controls) of 0.1 (a value comparable with those used by previous studies [56,57]), we also observed similar proportions of differential expression as we observed in this study. Moreover, genes identified as differentially expressed in our initial set of 174 samples also tended to be differentially-expressed in an independent set of 42 samples. These results suggest the identified novel lncRNAs in this study are true positives with high confidence. Furthermore, using genes with high skin specificity (*T*_*s*_ >0.4), we observed higher proportions of differential expression in psoriatic skin for protein-coding genes (46%) and annotated lncRNAs (38%), when comparing with results from Table 2: 17% for protein-coding genes and 24% for annotated lncRNAs. In concordance with the observation that novel lncRNA has high skin-specificity and proportion of differential expression, these findings suggest that skin specificity is an important factor contributing to the dysregulation of transcription in psoriatic skin.

In our previous study [22], we showed the decrease in expression of dermal-specific genes could be due to the relative decrease in the dermal compartment in psoriatic skin when compared to normal skin [58]. We acknowledge the fact that difference in cellular compositions (for example, decrease in relative abundance of dermal cells or infiltration of immune cells) could play a role in yielding some differentially expressed genes when comparing the psoriatic and normal skin. The laser-capture microdissection experiments we used in our previous study to identify the epidermal- and dermal-specific genes are microarray-based. This limited our ability to evaluate whether the differentially expressed novel lncRNAs are cell intrinsic or due to the change in cellular proportions within skin tissue. However, our work has generated important hypotheses and questions regarding the study of transcriptomic architecture in complex tissues. Future studies measuring gene expression for specific cell types captured by fluorescence-activated cell sorting and/or laser capture microdissection will be able to facilitate the assessment and evaluate whether the differentially expressed lncRNAs in skin tissues are cell intrinsic or due to the change in cellular proportions.

One genomic region known to contain many genes that are selectively expressed in skin is the EDC [31,39,40,59]. Indeed, we found that the EDC was among one of the genomic regions of highest lncRNA densities (Figure 2), with significant enrichment for novel lncRNAs (12 novel out of 28 total, *P* = 3 × 10^−2^). In contrast, the other high lncRNA density region, the MHC, showed no significant enrichment (6 novel out of 26 total, *P* = 0.92). While the MHC does contain the corneodesmosin (*CDSN*) gene, which is relatively specifically expressed in skin and hair, it is not generally enriched in genes specifically expressed in skin. We acknowledge the fact that the MHC contains several hundred genes [1,41] and it is more challenging to identify novel lncRNAs in this area after imposing the ‘distance to known annotated genes’ filter (Figure 1). However, abandoning this filter would increase the identification of artefactual novel lncRNAs arising to immature transcripts from previously annotated genes.

As observed for many other complex diseases [60,61], the majority of genetic susceptibility loci identified for psoriasis fall into non-coding regions [42,62,63]. These findings challenge us to identify the non-coding elements in these regions which might be responsible for conferring susceptibility. One possible scenario is that genetic variants (expression quantitative trait loci, or eQTLs) mapping to the enhancer/promoter regions could play important roles in regulating gene expression levels in different tissues [64]. Indeed, we have shown enrichment for psoriasis susceptibility signals in psoriasis eQTLs, relative to non-eQTLs [65]. However, variations in lncRNAs should also be taken into account. The fact that we observed similar ratios of expressed lncRNAs (103 out of 4,022: 2.6%) and protein-coding mRNAs (450 out of 14,011: 3.2%) within the susceptibility loci suggest the identification and interpretation for causal elements for disease association should not be restricted to protein-coding transcripts. Indeed, single-nucleotide variations in lncRNAs (‘riboSNitches’) have been shown to map to eQTLs in disease susceptibility regions, suggesting that they may directly confer risk for complex traits [48], and are strong candidates for further investigation.

## Conclusions

In conclusion, we have identified a substantial number of skin-specific lncRNAs in this study, and we have imputed potential immunological functions for them in the pathogenesis of psoriasis. Moreover, our results provide interesting potential clues into the mechanisms of tissue-specific gene regulation. As the roles of lncRNAs in other human autoimmune diseases have not yet been fully identified and understood, this analysis should provide valuable resource and information for the future studies.

## Materials and methods

### RNA-seq samples

The preparation methods and quality control procedures used for the initial set of 174 RNA-seq samples (92 PP, 82 NN) have been described [22], and the same protocol was used for the additional 42 RNA-seq samples (7 PP, 8 NN, and 27 PN). In order to limit the variability of expression caused by treatment, we required a washout period prior to biopsy: at least 1 week for topical medications and 2 weeks for phototherapy/systematic medications. Informed consent was obtained from all subjects under protocols approved by the University of Michigan Institutional Review Board (HUM00037994) and adheres to the Declaration of Helsinki Principles.

### Identification of unannotated transcripts

We used Tophat [25] (version 1.3.3) to perform alignment and Cufflink [26] version 2.1.1 to perform *ab initio* transcript assembly. We then estimated read counts for each identified genes using the ReadCount software [66]. We then employed an enhanced version of a computational approach [13] which combined the information across the samples in the dataset to detect any unannotated transcripts using gene models from Ensembl version 74 as reference [27]. The identified transcripts were classified in eight different categories (Additional file 2: Table S2). We used the presence of AT/GU splice site sequences to predict the strand orientation for transcripts with at least 2 exons.

We applied different filtering procedures to remove potential artifacts for novel transcripts identified. We first required that transcripts have coverage in at least 5% of the samples in our dataset (that is, ≥11 samples). To remove the unannotated transcripts that may be fragments of premature mRNA from annotated genes, we estimated for each annotated gene *i* the distance (*d*_*i*_) to the exon of another annotated gene. We then used the median distance of annotated lncRNA to determine the distance threshold (<2 kb) for removing potential premature mRNA fragments. We acknowledge that this criterion will remove true positive novel lncRNAs (assuming the same distribution of *d*_*i*_ for annotated and novel lncRNAs), but in this study we imposed stringent criteria to reduce false positive results and as shown in Table 1 this distance filter removes more than half of the unannotated transcripts in some categories of unannotated transcripts. Sequence reads mapped to regions of low mappability could generate potential false positive transcripts; we obtained the uniqueness (35-mers) and alignability (75-mers) tracks from the UCSC Genome Browser [67] and computed the gene-wise mappability measure (that is, uniqueness and alignability scores) using the bigWigSummary tool from the UCSC Genome Browser. We retained novel transcripts with score of at least 0.9 in both mappability measures; for comparison, 80% and 88% of annotated ncRNAs transcripts have scores of ≥0.9 for the uniqueness and alignability measures, respectively. Next, we applied a minimum transcript length criterion (≥200 bp) to remove short transcripts.

### Evaluation of novel lncRNAs

We evaluated the coding potential of the identified novel lncRNAs using TransDecoder [68] and txCdsPredict from UCSC Genome Browser. For the prediction using txCdsPredict, a score greater than 800 was used as a criterion (90% predictive of protein coding genes [13]). Additional file 3: Figure S7 shows the percentage of genes predicted to be candidates of coding transcripts by different approaches. Only two of the identified novel lncRNAs were predicted to have coding potential by both approaches.

### Expression analysis

We used DESeq, which implements a read count model based on negative binomial distribution, to perform the expression normalization and differential expression analysis [30] for three different comparisons: (i) lesional psoriatic (PP) versus normal skin (NN); (ii) PP versus paired uninvolved (PN) skin from 27 psoriatic patients; and (iii) uninvolved (PN) versus NN skin. Significantly differentially expressed genes were declared to have FDR ≤0.1 and |log_2_ fold change| ≥1.

### qRT-PCR analysis

qRT-PCR analysis of selected genes was performed using six lesional skin samples from psoriasis patients (PP), six uninvolved skin from psoriasis patients (PN), and six normal skin from control subjects (NN). These samples were independent of the samples we used in the RNA-seq experiments. Skin biopsies were flash-frozen in liquid nitrogen and stored at 80°C. RNA extractions were performed using RNeasy columns (Qiagen, Cat # 74136). RNA quantity and quality were measured on an Agilent 2100 Bioanalyzer (Agilent Technologies), and only samples yielding intact 18S and 28S ribosomal RNA profiles were used. Reversed transcription was performed using High Capacity cDNA Transcription kit (Applied Biosystems, Cat # 4368813). Transcripts were quantified by SYBR green fluorescence (Applied Biosystems, Cat # 4367659) using 7300 Real-Time PCR system (Applied Biosystems). Relative expression was quantified using large ribosomal protein P0 (*RPLP0*) as an internal reference. In the qPCR process, primers for G36220, 5′-AGG ATG TTC CCC TGC TTT TT-3′ and 5′-CAC TCT TGC GAT GAA GTG ATG-3′; for G25746, 5′-CCC CTG AGA CAT TTC TTC CA-3′ and 5′-AGC CTT GGA GGG TTT CAA AT-3′; for G2608, 5′-GGC CTT ATC TTT TGC ACC TG-3′ and 5′-CAA CCA GCC AAA TTC CTG TT-3′; and for RPLP0, 5′- GCT GAT CCA TCT GCC TTT GT-3′ and 5′- AAG TTG GTT GCT TTT TGG TGA-3′. All custom primers were purchased from Sigma-Aldrich.

### Evaluation of tissue specificity

We downloaded RNA-seq sequence data from two independent RNA-seq cohorts [23,33] from the NCBI Gene Expression Omnibus [69], and performed alignment by Tophat using the same parameters and arguments as we described above. The Body Map 2.0 project [33] consists of 16 different tissues, and the other study [23] consists of three pairs of PN/PP skin samples. We then measured the expression level in these RNA-seq datasets for each gene we identified in this study. The tissue specificity (*T*_*s*_) of a gene in tissue *s* was calculated as the fraction of expression (in RPKM) relative to the sum of its expression in all 17 tissues. We averaged the RPKM values between the three PN samples in the Jabbari *et al.* study to estimate the skin’s RPKM values.

We downloaded the chromatin state segmentation [34,35] files for nine different cell lines (GM12878, H1-hESC, K562, HepG2, HUVEC, HMEC, HSMM, NHEK, and NHLF) from the UCSC Genome Browser, and retrieved the predicted strong enhancer and active promoter elements for each cell line. For each gene, we computed the distance to the closest enhancer/promoter from the starting position of the gene. To evaluate whether the enhancer/promoter regions are closer to novel lncRNAs in ectodermally-related cell types (NHEK and HMEC) than in other cell types, we computed the relative distance (*D*_*ecto*_*/D*_*average*_), where *D*_*ecto*_ is the closest distance to the enhancer (or promoter) in ectodermally-related cell type (NHEK or HMEC), and *D*_*average*_ is the average closest distance to the enhancer (or promoter) in the other cell types. The means and standard error bars from Figure 5 were computed after removing the outlier distances (that is, 1.5 times the inter-quartile range).

### Functional characterization of the identified lncRNAs

We first examined if any of the expressed transcripts are within the previously identified regions of psoriasis susceptibility loci [42]. The associated regions were defined by ±500 kb intervals (±3 Mb for MHC) with respect to the best genome-wide significant signal.

Next, we obtained the functional and pathway annotation data from the GO [43], KEGG [44], and Reactome [70] databases. We further processed GO’s gene-to-GO file to annotate each gene with all the ‘ancestral’ terms of its annotated term(s) in the directed acyclic graph of GO database. We inferred the functions of each lncRNA using the biological functions/pathways for annotated the most correlated gene(s). We first evaluated the number of lncRNAs with inferred functions using different minimum squared Spearman correlation coefficient cutoff for the most correlated gene(s) (Additional file 3: Figure S14). To determine an optimal minimum correlation cutoff for inferring functions of lncRNAs, we applied a sampling method to co-expression patterns of functional annotated genes. Our sampling approach obtained the maximum correlation between a randomly selected gene with the other genes annotated in the same function versus with genes not annotated in the same function, and it considered the probability that two genes would be annotated in the same pathway. We performed 100,000 samplings. For each sample, we randomly selected two genes expressed in our dataset; if the two genes belonged to the same annotated function, we then randomly picked one gene and retrieved the maximum correlation between that gene with any other genes in the same function. If the two genes did not belong to the same function, we then randomly picked one gene and calculated the maximum correlation with other randomly selected genes. The number of randomly selected genes would be determined by the distribution of the number of genes in each annotated function. By using this approach, we obtained an area under the receiver operating characteristic curve = 0.73 when predicting if the most correlated gene is from the same function (Additional file 3: Figure S13). We then computed the precision (*ρ*: proportion of significant gene pairs from same functions) and recall (*ϒ*: proportion of gene pairs from the same functions that are significant) under different minimum correlation cutoffs to obtain F-measures (Additional file 3: Figure S15):

$$ {F}_{\beta}\kern0.5em =\kern0.5em \left(1+{\beta}^2\right)\frac{\rho \gamma }{\left(\rho {\beta}^2\right)\kern0.5em +\kern0.5em \gamma } $$

where different *β* values would give different emphasis on precision/recall. We used the F-measure which emphasizes the recall (*F*_*β=5*_) in order to provide biological inference for larger number of lncRNAs, and used the correlation cutoff that maximizes the F-measure.

### Preparation and analysis of keratinocyte RNA-seq libraries

Normal human epidermal keratinocytes prepared from adult skin as described [71] were grown to post-confluence as described [72], prior to addition of recombinant human IL-17 and/or TNF (each at 10 ng/mL). After 24 h of treatment, total RNA was isolated using Qiagen RNeasy Minikits (Valencia, CA, USA) and RNA quality and quantity were assessed using an Agilent Bioanalyzer. Libraries for high throughput sequencing were prepared using the Illumina mRNA-Seq kit according to the manufacturer’s description (Illumina, San Diego, CA, USA) and libraries were sequenced on the Illumina Genome Analyzer IIx. The alignment and expression quantification procedures were identical to those described above. We performed differential expression analysis using DESeq. Among genes with a differential expression FDR ≤0.1, those with a FC >2 were declared as significantly enhanced and those with a FC <0.5 as significantly repressed.

### Data availability

The RNA-seq data used for this analysis are accessible through GSE63980 (superseries of GSE54456 and GSE63979).

## Additional files

Additional file 1: Table S1. Summary of the RNA-seq cohort. Additional file 2: Table S2. Categorization of transcripts identified. Additional file 3:
**Figures S1-15.**
Additional file 4: Table S3. Genes passed all quality control and filtering steps and used in the analysis. Additional file 5: Table S4. Differentially expressed identified by DESeq in three comparisons. Additional file 6: Table S5. The gene IDs for the elncRNAs and plncRNAs. Additional file 7: Table S6. Catalog of expressed lncRNAs in psoriasis susceptibility loci. Additional file 8: Table S7. lncRNAs with inverred functions. Additional file 9: Table S8. Enrichment results for the inferred functions in the differentially expressed lncRNAs. Additional file 10: Table S9. Cytokine-enhanced and cytokine-repressed lncRNAs. Additional file 11: Table S10. Observed-to-expected ratios for the number of cytokine-induced genes which were upregulated in PP.

## Abbreviations

DEGs: Differentially expressed genes
elncRNA: Enhancer-associated long non-coding RNA
FC: Fold change
LD: Linkage disequilibrium
lncRNA: Long non-coding RNA
NN: Normal skin
plncRNA: Promoter-associated long non-coding RNA
PN: Uninvolved skin
PP: Psoriatic skin
RNA-seq: High-throughput cDNA sequencing
