# Supplementary material for: Analysis of long non-coding RNAs highlights tissue-specific expression patterns and epigenetic profiles in normal and psoriatic skin
Source: Genome Biol. 2015 Jan 30;16(1):24. doi: 10.1186/s13059-014-0570-4 (PMC4311508; doi:10.1186/s13059-014-0570-4)
Supplement: Additional file 3: — Figures S1-15. [file 13059_2014_570_MOESM3_ESM.docx]

**Supplementary Information**

**Supplemental Figures**

**
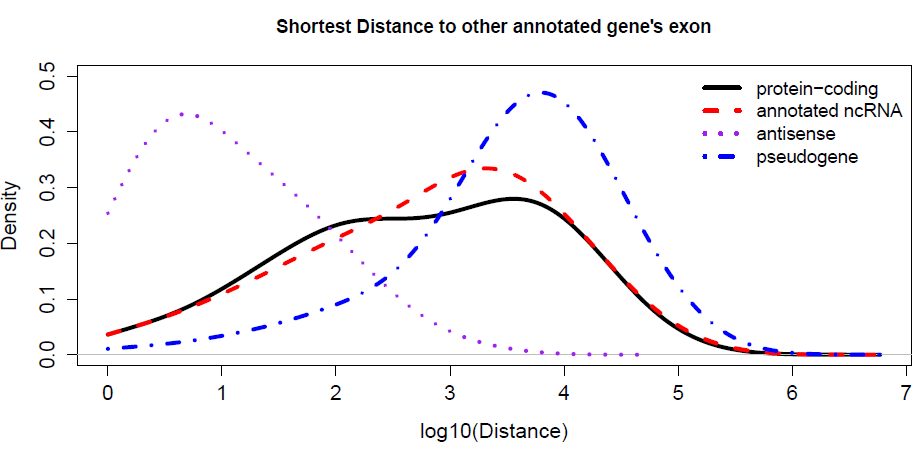
**

**Figure S1. Genomic distance to the closest exon of neighboring annotated genes.**

**
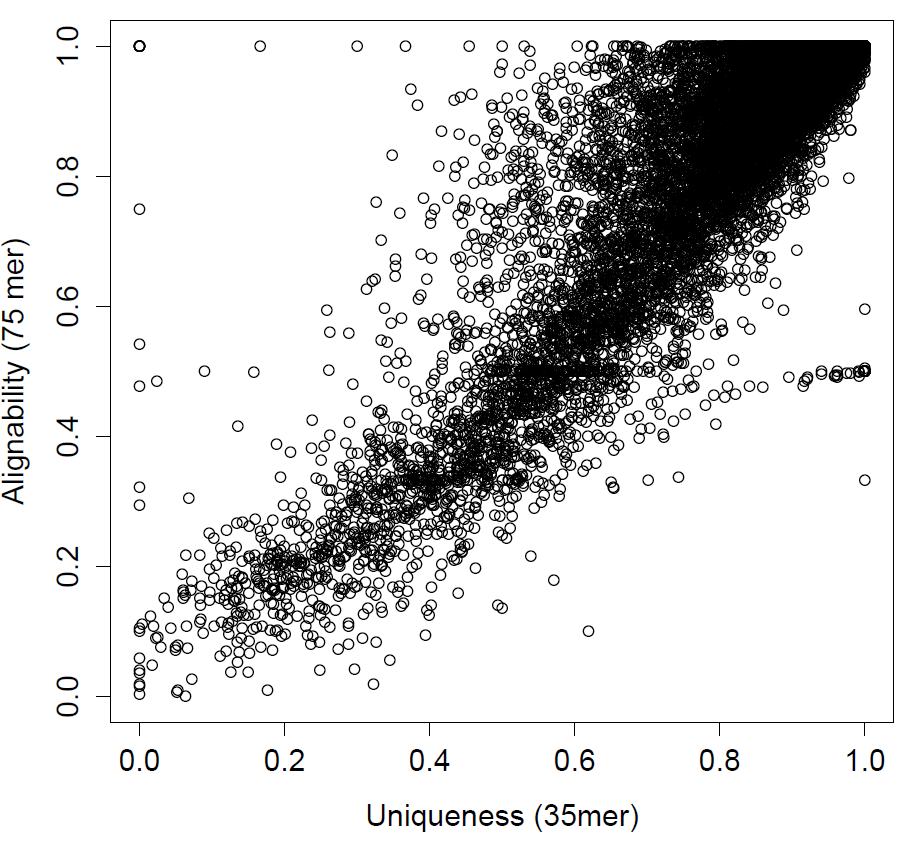
**

**Figure S2. Average alignability (75bp) and uniqueness (35bp) scores for each identified transcript.**

**
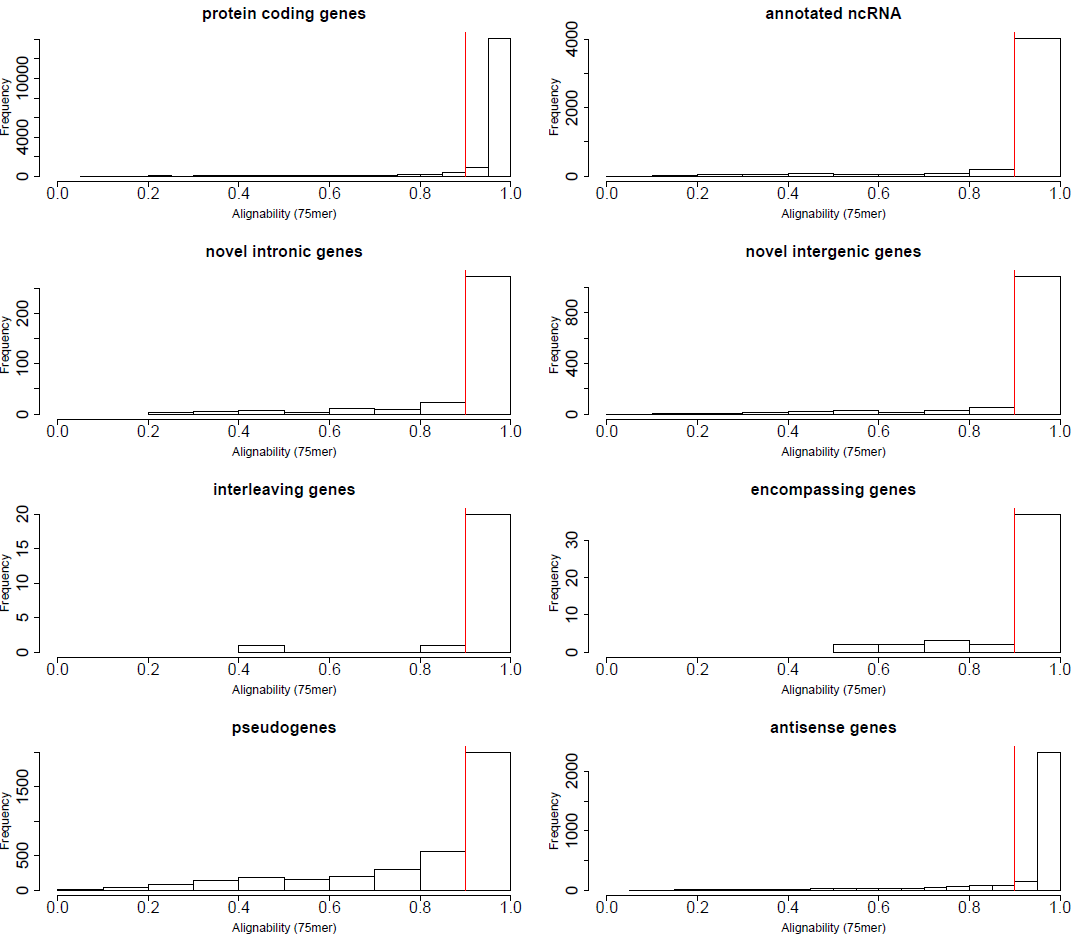
Figure S3. Histogram distribution for average alignability score of transcripts under different categories.**

**
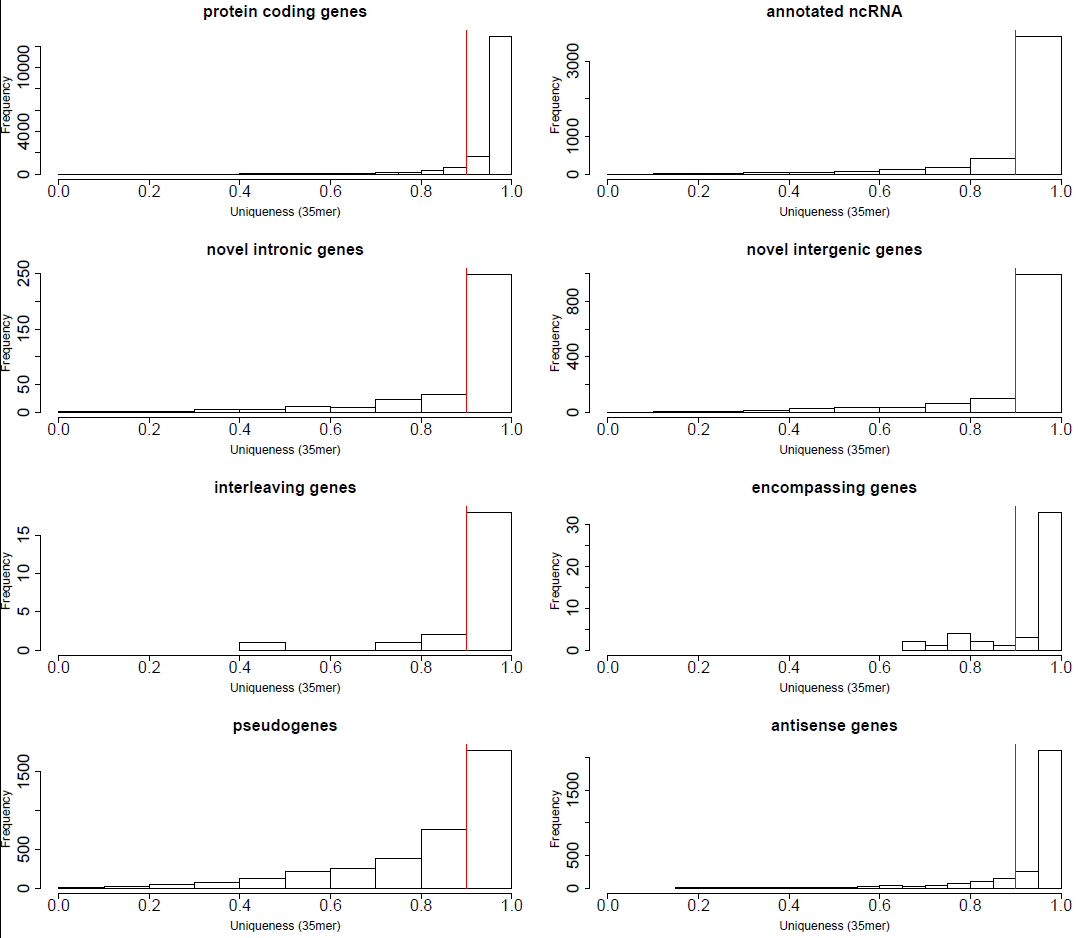
**

**Figure S4. Histogram distribution for average uniqueness score of transcripts under different categories.**

**
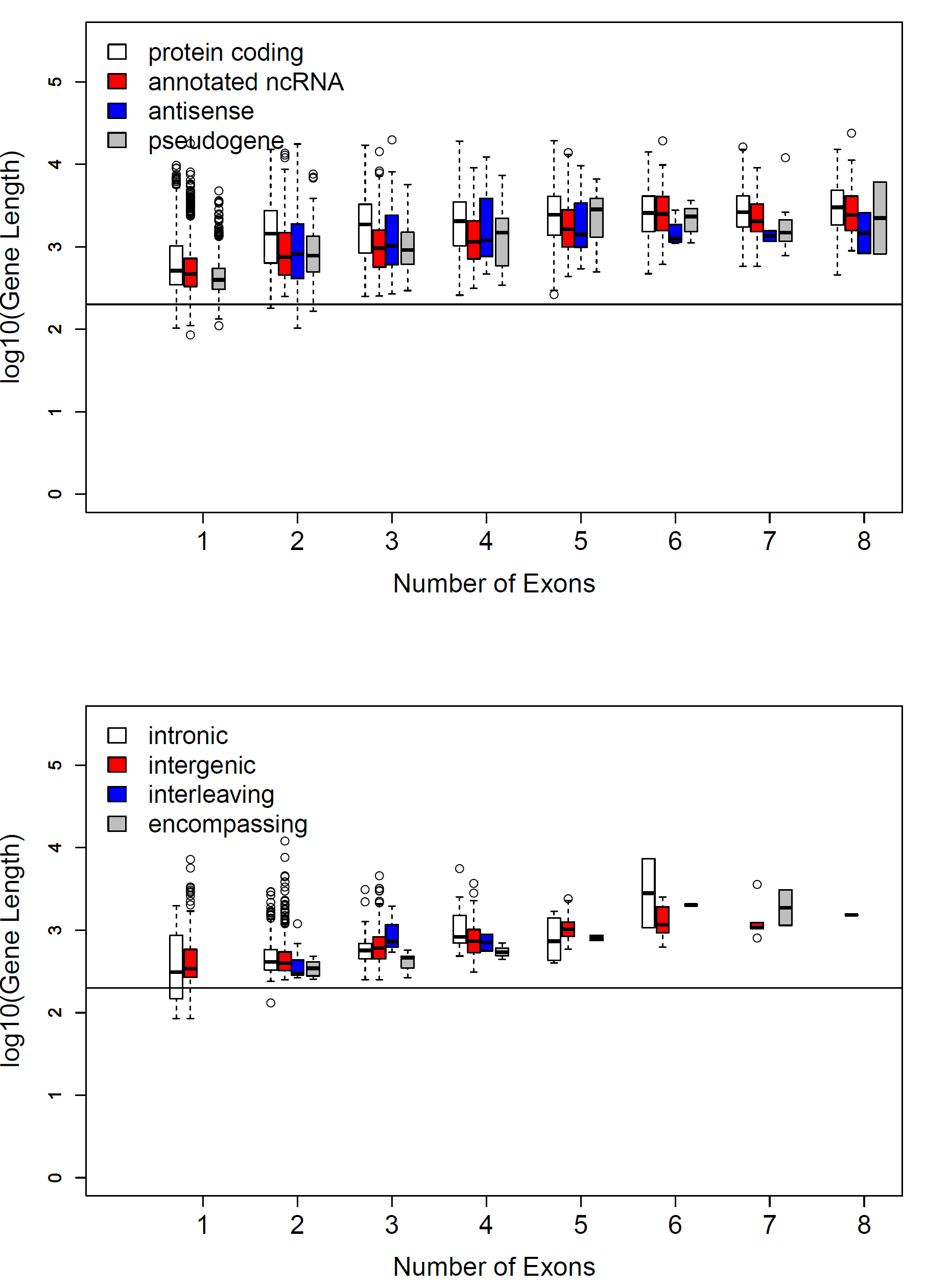
**

**Figure S5. Transcript length (in logarithmic scale) of different gene categories as a function of the number of exons. Top and bottom panels show the annotated (or known) transcripts and novel transcripts, respectively.**

**
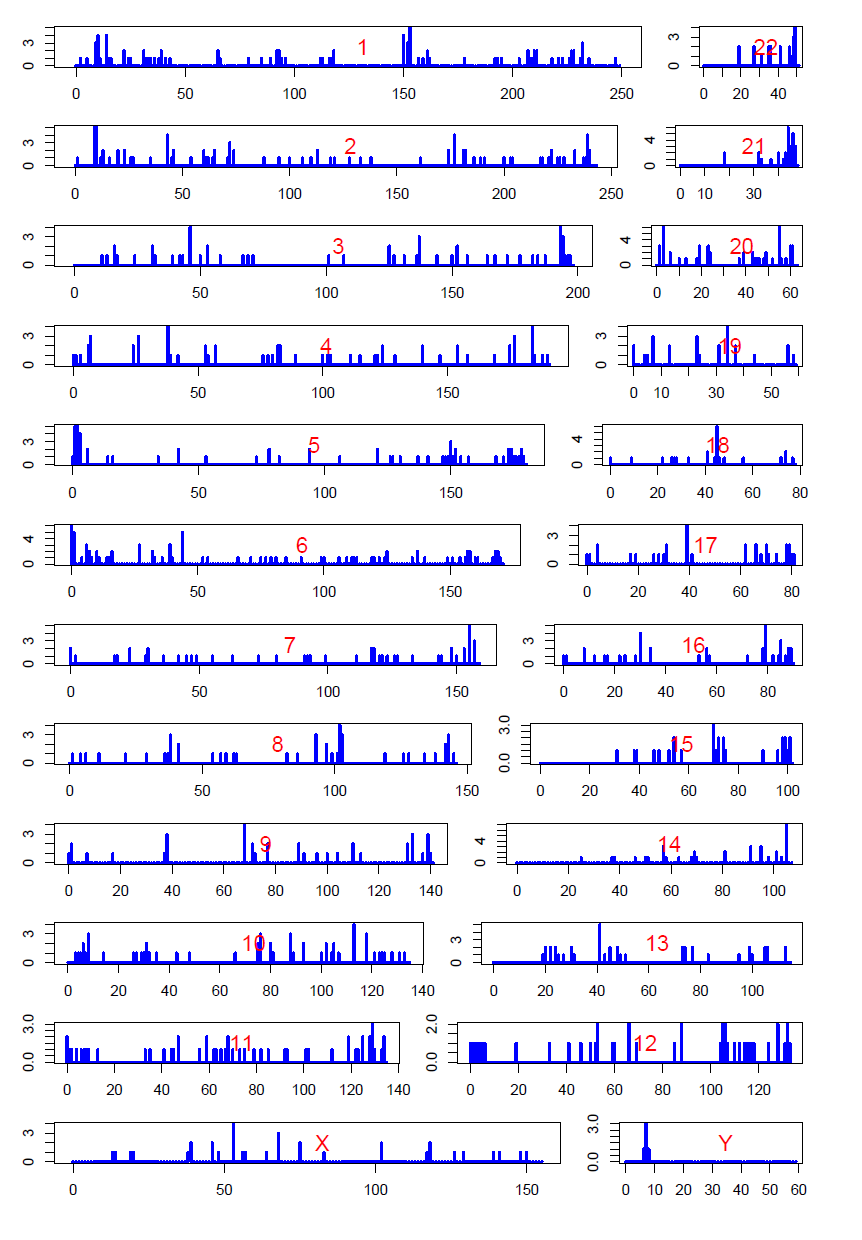
**

**Figure S6. Density map of identified novel lncRNAs expressed in skin tissues across the genome.**


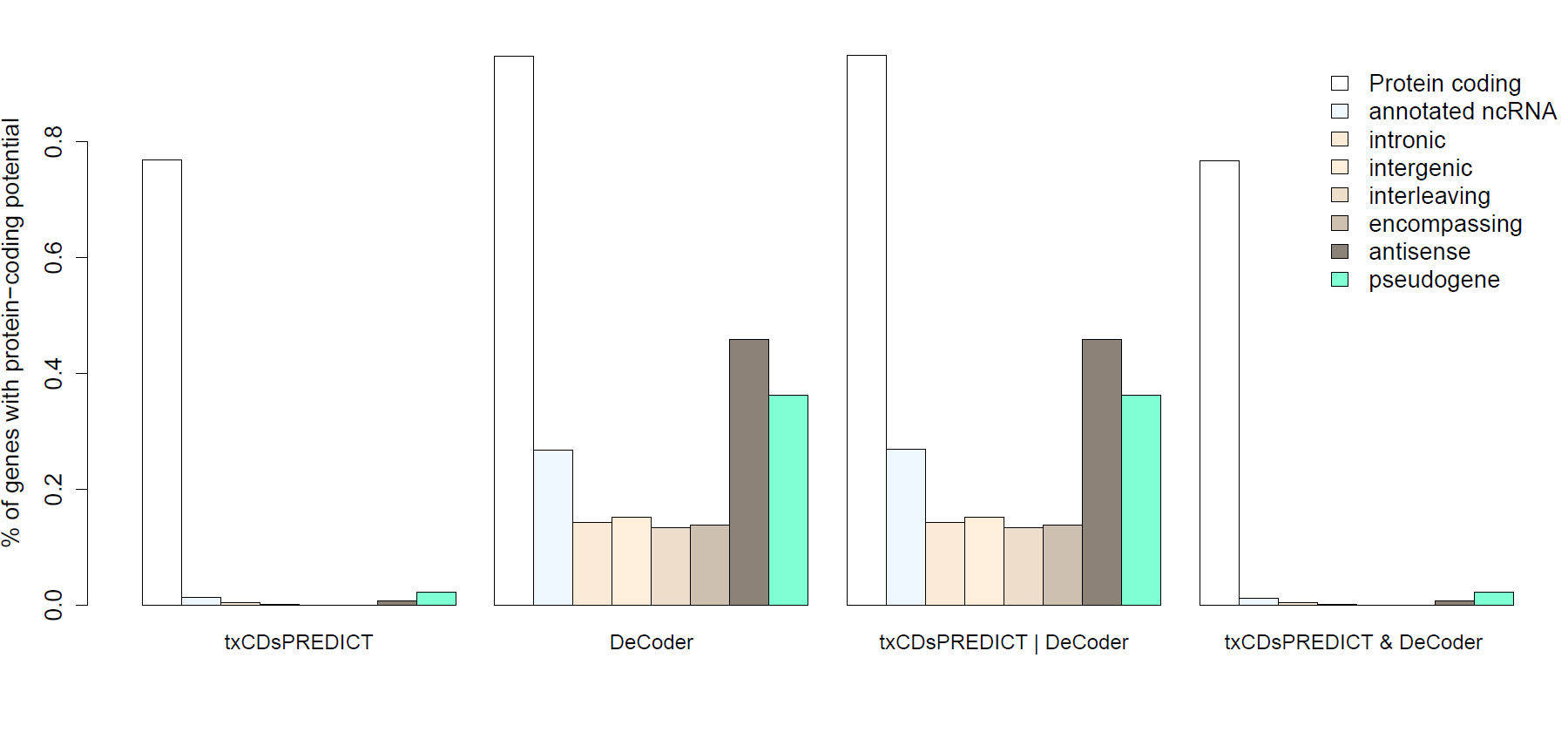


**Figure S7. Percentage of genes predicted to have coding potential by different approaches: predicted by txCDs only; DeCoder only; txCDs or DeCoder; txCDs and DeCoder.**

**
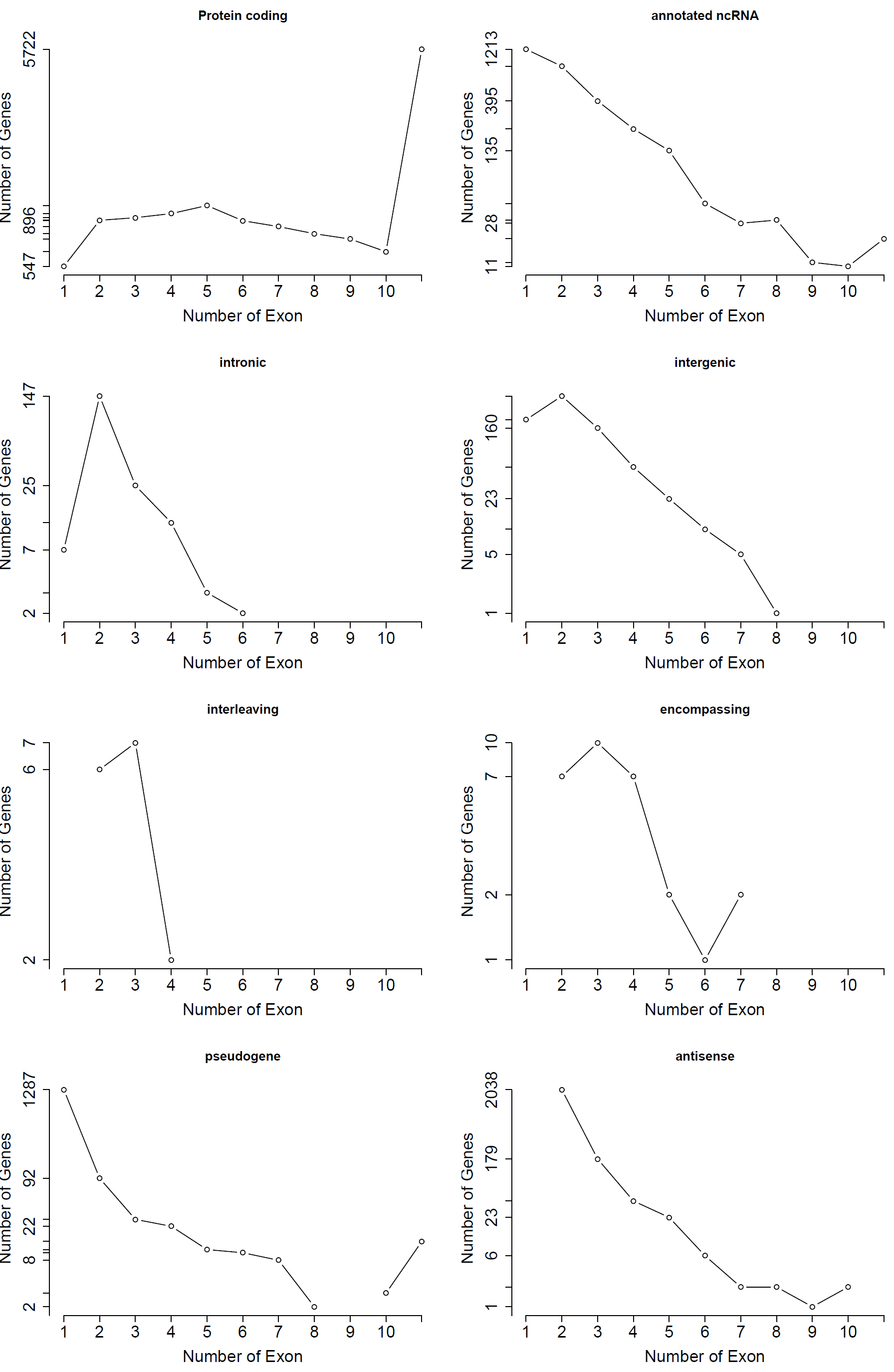
**

**Figure S8. Frequency distribution for the number of exons of a gene for the eight different categories.**

**
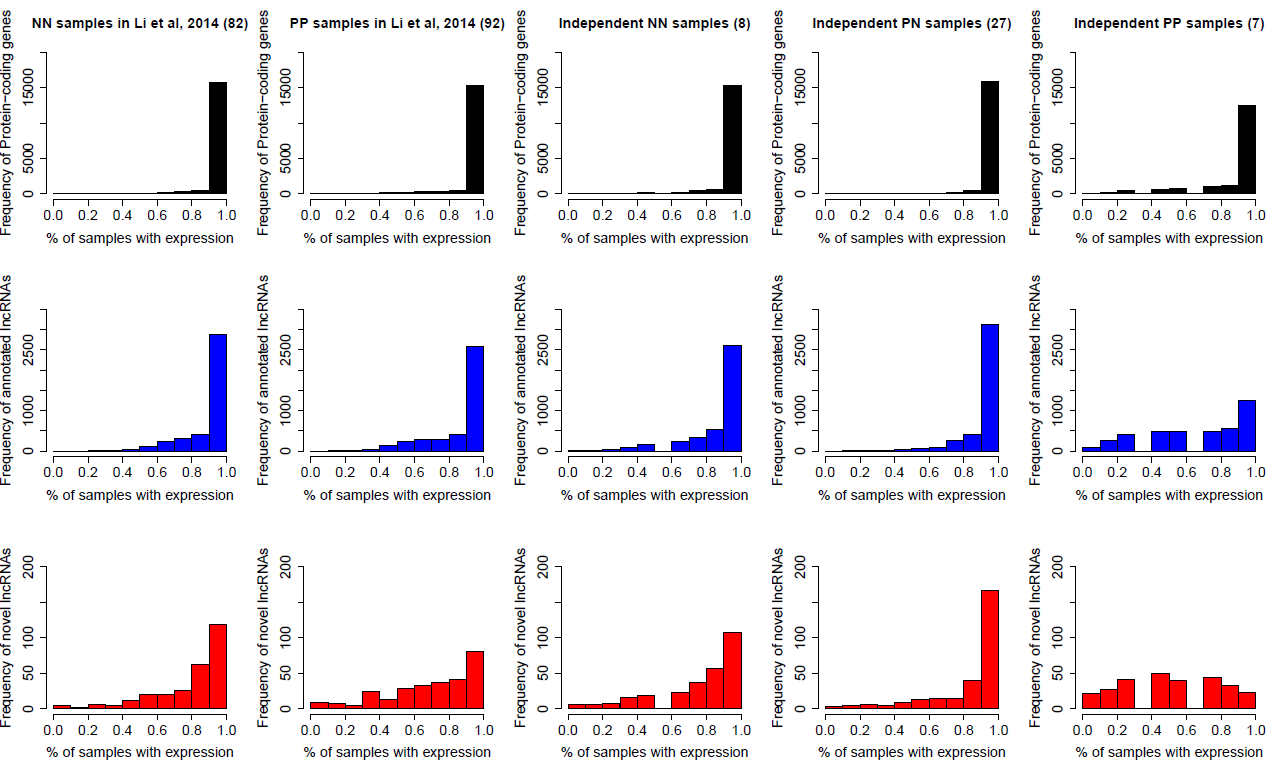
Figure S9. Proportions of genes expressed in the discovery dataset of 174 samples (left two columns) and in the independent dataset of 42 samples (right three columns). The numbers in the brackets indicate the number of samples used in each dataset.**

**a**

**
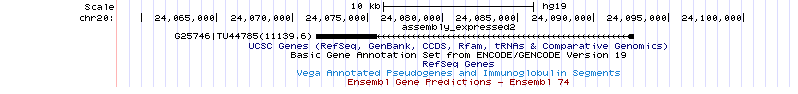
**

**b**

**
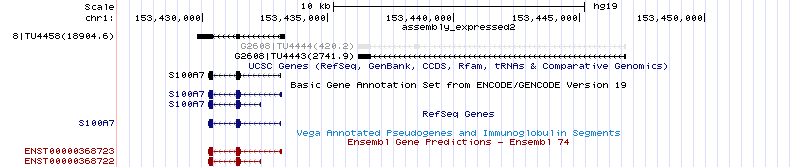
**

**Figure S10. The most downregulated (a) and upregulated (b) novel lncRNAs in psoriatic skin.**

**
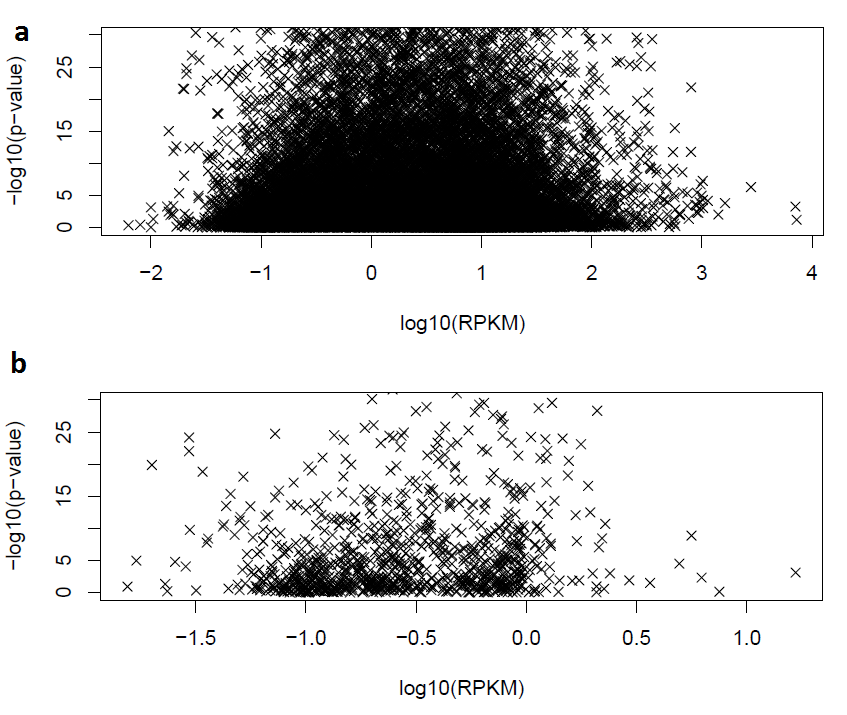
**

**Figure S11. Differential expression *P* value versus median logarithmic RPKM levels for annotated genes (a) and novel lncRNAs (b). The *P* values of Spearman tests for examining the negative associations between the negative logarithmic differential expression *P* values versus the logarithmic RPKM values (H_0_: no association or positive association) are 1 and 0.937.**

**Figure S12. qrt-PCR results for three lncRNAs under different conditions (NN, PN, and PP), where six independent samples were measured for each condition (mean and standard deviation are shown in the Figure). Consistent with the RNA-seq results, the three genes both show significant (*P* <0.05) differential expression in psoriatic skin: G2608 (NN vs. PP: *P* = 3.7 × 10^-2^; PN vs. PP: *P* = 3.7 × 10^-2^); G25746 (NN vs. PP: *P* = 6.2 × 10^-3^; PN vs. PP: *P* = 2.2 × 10^-4^); and G36220 (NN vs. PP: *P* = 1.7 × 10^-2^; PN vs. PP: *P* = 8.4 × 10^-2^).**

**
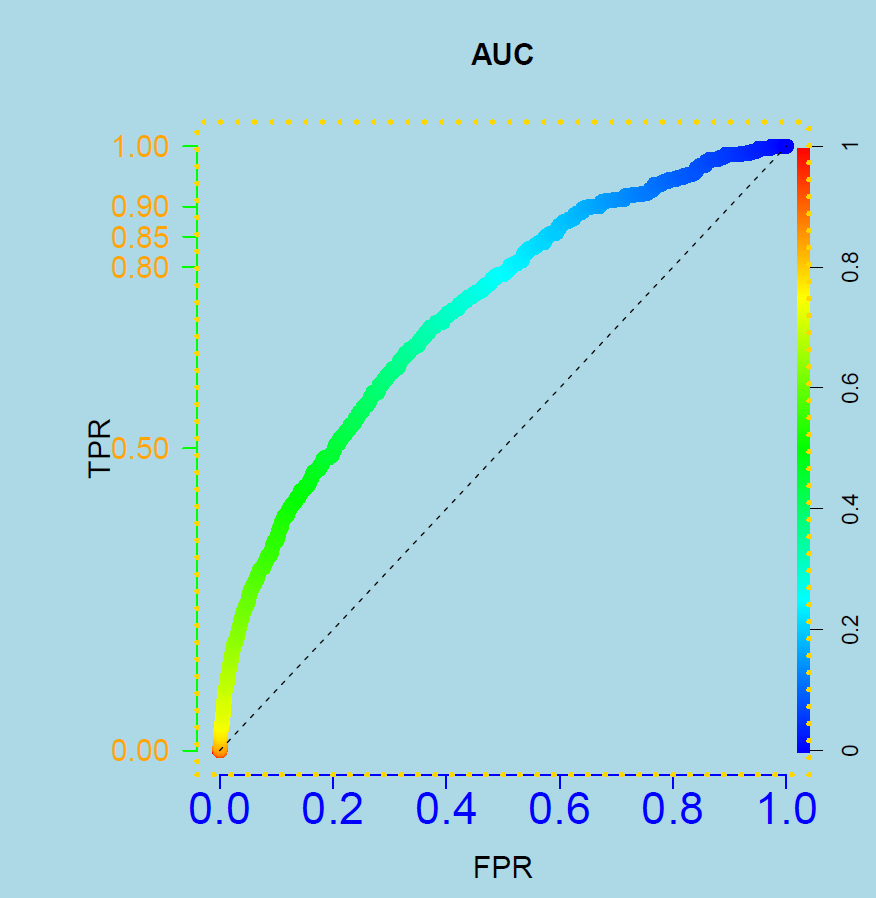
**

**Figure S13. Receiver operating characteristic (ROC) curve for using the most correlated gene to predict if a protein-coding gene pair is annotated in the same function/pathway under different gene expression correlation cutoffs. Area under the ROC = 0.73.**


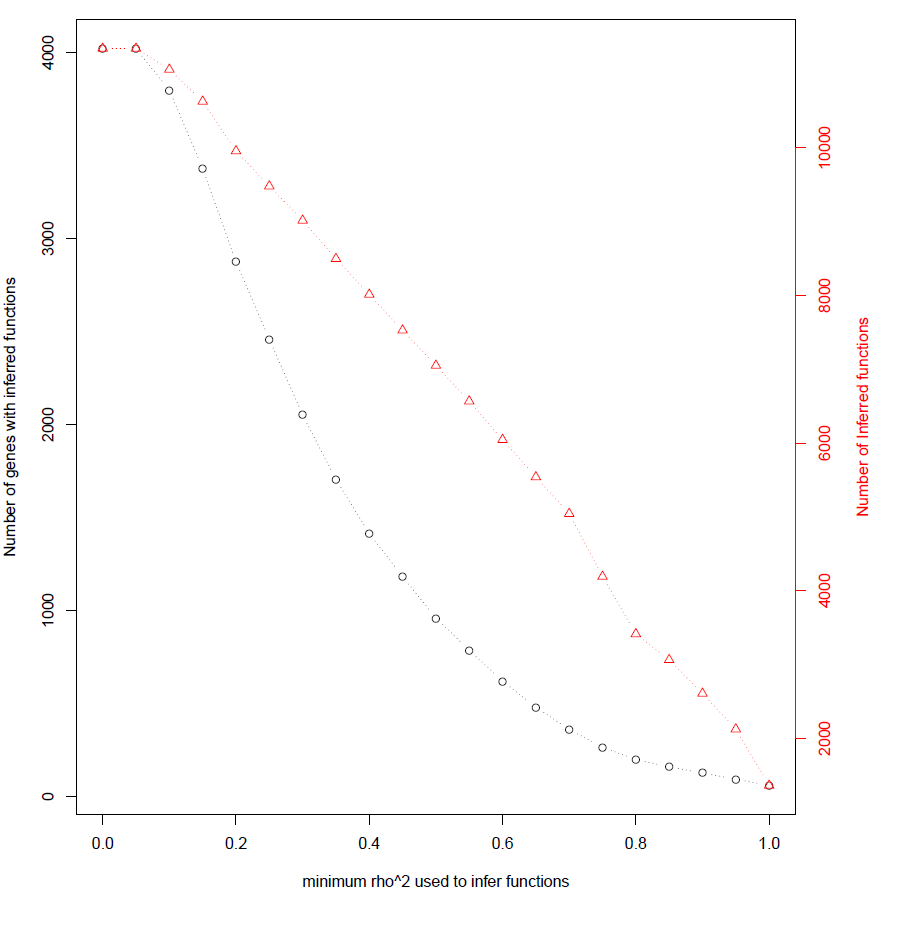


**Figure S14. Number of lncRNAs with inferred biological functions/pathways versus different minimum squared spearman coefficient used in the co-expression analysis. The y-axis on the right (red) indicates the number of inferred functions for those lncRNAs.**


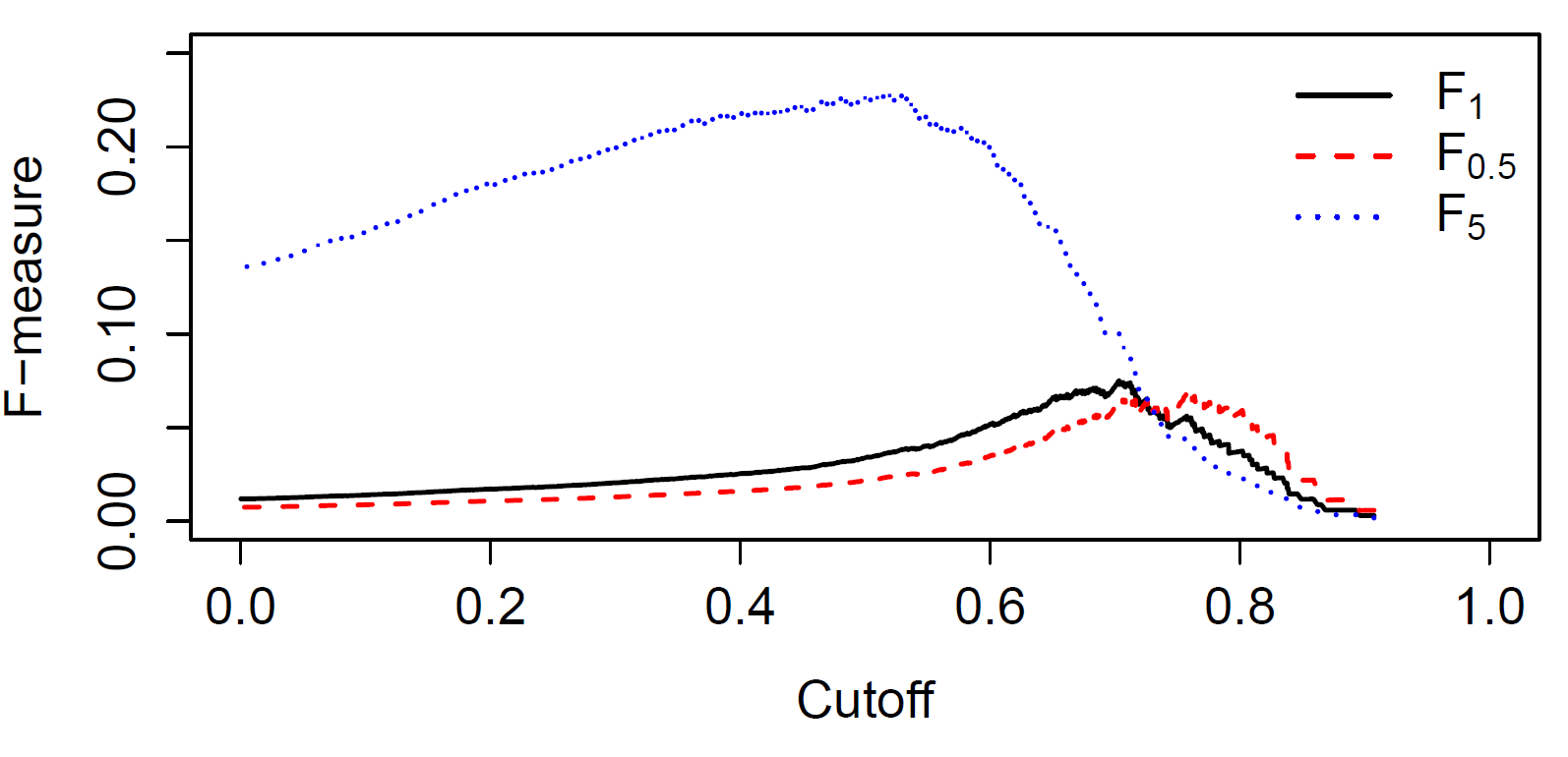


**Figure S15. F-measures values under different squared spearman coefficient minimum cutoff. Different F-measures computed using different contribution from the precision and recall when using the most correlated gene to predict if a gene pair is involved in the same biological function/pathway. We used the F-measure which emphasizes the recall, and obtained the squared spearman coefficient cutoff (approximately 0.5) which optimized it.**
